# Theoretical prediction of nanosizing effects and role of additives in the decomposition of Mg(BH_4_)_2_†

**DOI:** 10.1039/d3ra08710g

**Published:** 2024-02-20

**Authors:** Stefano Pantaleone, Elisa Albanese, Lorenzo Donà, Marta Corno, Marcello Baricco, Bartolomeo Civalleri

**Affiliations:** a Dipartimento di Chimica and NIS Interdepartmental Centre, Università degli Studi di Torino via P. Giuria 7 10125 Torino Italy bartolomeo.civalleri@unito.it

## Abstract

The energetic transition towards renewable resources is one of the biggest challenges of this century. In this context, the role of H_2_ is of paramount importance as a key source of energy that could substitute traditional fossil fuels. This technology, even if available in several manufactures, still needs to be optimized at all levels (production, storage and distribution) to be integrated on a larger scale. Among materials suitable to store H_2_, Mg(BH_4_)_2_ is particularly interesting due to its high content of H_2_ in terms of gravimetric density. Nanosizing effects and role of additives in the decomposition of Mg(BH_4_)_2_ were studied by density functional theory (DFT) modelling. Both effects were analyzed because of their contribution in promoting the decomposition of the material. In particular, to have a quantitative idea of nanosizing effects, we used thin film 2D models corresponding to different crystallographic surfaces and referred to the following reaction: Mg(BH_4_)_2_ → MgB_2_ + 4H_2_. When moving from bulk to nanoscale (2D models), a remarkable decrease in the decomposition energy (10–20 kJ mol^−1^) was predicted depending on the surface and the thin film thickness considered. As regards the role of additives (Ni and Cu), we based our analysis on their effect in perturbing neighboring borohydride groups. We found a clear elongation of some B–H bonds, in particular with the NiF_2_ additive (about 0.1 Å). We interpreted this behavior as an indicator of the propensity of borohydride towards dissociation. On the basis of this evidence, we also explored a possible reaction pathway of NiF_2_ and CuF_2_ on Mg(BH_4_)_2_ up to H_2_ release and pointed out the major catalytic effect of Ni compared to Cu.

## Abstract 2

Nanosizing effects and role of additives in the decomposition of Mg(BH_4_)_2_ were studied by DFT modelling. Decomposition is favoured at the nanoscale with respect to the bulk along with a major catalytic effect of Ni compared to Cu.

## Graphical abstract

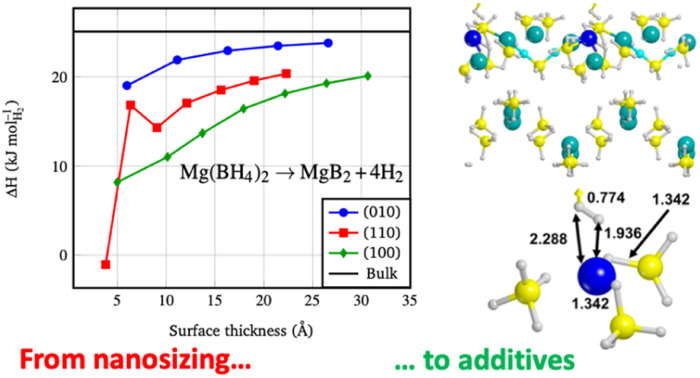

## 1 Introduction

Since many decades, the response to the more and more increasing energetic demand has focused its attention on the optimization of existing industrial processes, on their environmental impact, and on the development of new sustainable techniques. In this context, the hydrogen economy plays a key role,^1–8^ because hydrogen is abundant on the earth (obviously not in its molecular form) and the only product of the reaction of a fuel cell is water, with no byproducts that may cause environmental pollution;^9^ in particular, one of the most important research lines is to find an efficient way to store it.^10,11^ The first fuel cell vehicle prototype was developed in 1966 by General Motors,^12^ but only in 2004 hydrogen was used for public transportation in the city of Stockholm,^13^ and only in 2008 Honda built the first car for private usage.^14^ In the recent years this technology is becoming more and more available for daily uses,^15,16^ but still needs to be optimized to be cost-effective in comparison to classical fossil fuels.

The research is focusing its attention on all the stages of this technology: the H_2_ production, its storage and distribution, and an efficient way to make it cost-competitive and available for the most common usage (automotive and whatever means of transport, industrial, residential, *etc.*). In the present study we will focus in particular on its storage.

The principal ways to store hydrogen are as: pressured gas, cryogenic liquid, and using carriers (liquid organic molecules or solid metal and complex hydrides) or confinement/adsorption in microporous scaffolds as metal–organic frameworks (MOF). Since compressed gas and cryogenic liquid present several problems related to efficiency, hydrogen storage using solid carriers represents a more suitable choice. Indeed, several materials present high concentration of hydrogen, such as alanates (AlH_4_^−^), amides (NH_2_^−^), borohydrides (BH_4_^−^), but also simpler binary compounds (metal hydrides),^17^ which, by thermal decomposition, produce molecular hydrogen (H_2_).^18–22^ To be sustainable, the cost-efficiency trade-off of this novel technology must satisfy some parameters imposed by EERA (European Energy Research Alliance) and HER (Hydrogen Europe Research), whose targets, in term of gravimetric and volumetric hydrogen density, are 4–10 wt% and 80–150 g_H_2__ L^−1^, respectively.^23^ In recent years, Mg(BH_4_)_2_, among others, has been intensively studied^24–32^ because it contains a large amount of hydrogen in particular in terms of gravimetric density (14.9 wt%), also keeping the volumetric density (113.0 g_H_2__ L^−1^) acceptable.^25^ The above-mentioned feature refers in particular to the β-Mg(BH_4_)_2_ allotrope, which is the one of interest in the present work. However, borohydrides with high hydrogen content belonging to I and II group (alkali and alkaline earth metals as counterions of the borohydride group) often suffer from poor reversibility and high dehydrogenation temperatures.^33^ On the contrary, transition metal borohydrides are too unstable to be used as hydrogen storage materials, *i.e.* their decomposition is too favorable, and the re-hydrogenation process is difficult. There are many strategies to reach to best balancing between de-hydrogenation and hydrogenation cycles, such as formation of mixed borohydrides,^34^ inclusion of additives,^35–37^ nanostructuration^38–40^ and nanoconfinement.^39,40^

Specifically, in mixed borohydrides the stability of the bare material can be modified and tuned by combining different metals. Recent theoretical and experimental studies^41,42^ demonstrated that for pure α-Mg(BH_4_)_2_ the decomposition starts around 250 °C, while with a proper amount of ZnCl_2_ the decomposition temperature halves. Of course, the substitution is not limited to the cation, but also to the anion.^43^ A similar strategy is the inclusion of additives, which not only helps the decomposition, but also the reversibility of the reaction. In particular, transition metals-based additives have been proven to reduce the reactive conditions to promote the re-hydrogenation.^44–47^ Another method to promote hydrogen desorption is to take advantage of nanosizing effects, because it is well known in literature that nanoparticles have different properties with respect to the bulk, and in particular a lower decomposition energy.^48,49^ In a recent paper, it has been demonstrated that hydrated LiBH_4_ nanosheets release hydrogen up to 10 wt% at 70 °C.^50^ Nanoconfinement is a technique where porous materials (e.g. carbon nanotubes and MOF^51–55^) are used to synthesize nanoparticles of required sizes. It can be considered as an effect of nanostructuration, where the size of the nanoparticles is driven by the porosity of a certain material.^56^ Using this technique, a considerable reduction of the decomposition temperature of LiBH_4_ has been achieved (Δ*T* = 120 °C).^57^ Finally, it is also worth mentioning some recent works, where two of the above-mentioned strategies are applied at the same time. As an example, alkali^58^ and alkaline earth^59^ borohydride mixtures confined in Al porous scaffolds and in carbon nanospheres, respectively, have shown a good efficiency, both in terms of working temperature and re-hydrogenation cycle. Similarly, also the using of additives was combined with nanoconfinement,^60–62^ where, in some cases, the additive is used to change the properties of the scaffold.^63,64^

As abovementioned, Mg(BH_4_)_2_ still represents one of the best candidates for the optimization process of this technology. In particular, nanostructuring effects and the inclusion of additives, which we investigate in detail in this work, provided promising results,^65–67^ as also the combination of these two strategies.^68^

Here, we present a theoretical study of nanosizing effects and the role of additives in the decomposition of Mg(BH_4_)_2_ through first principles DFT calculations. Fully periodic boundary conditions were applied to all the model systems by using the CRYSTAL code. In the first part of the work we discuss the modelling of the decomposition of both bulk (3D) and surfaces (2D) to investigate nanosizing effects. In the second part, being inspired by the experimental work of Hauback and coworkers,^69^ we present the results of the doping of the bare 2D systems with different transition metals as possible additives to favor the decomposition of Mg(BH_4_)_2_.

Finally, we also studied the reaction pathway of the borohydride decomposition comparing the effect of two different metals: Ni and Cu.

## 2 Computational details

### 2.1 A model system for Mg(BH_4_)_2_

Magnesium borohydride presents several polymorphs that have been observed experimentally^70^ and other hypothetical phases that have been predicted theoretically;^71^ a few of them are listed in Table 1.

**Table 1:** Relative stability Δ*E* (eV/BH_4_) and density *ρ* (g cm^−3^) (*T* = 0 K, no zero-point-energy corrections) of different Mg(BH_4_)_2_ phases with respect to the *P*6_1_22 (α-phase) calculated by us at PBE-D* level, and as presented in ref. 76 and 77

Phases	*ρ*	*ρ*, ref. 76 and 77	Δ*E*	Δ*E*, ref. 76 and 77
*P*4_2_*nm* (δ)	0.997		0.128	
*Pmc*2_1_	0.984	0.934	0.053	0.066
*Fddd* (β)	0.800	0.790	0.046	0.056
*F*222	0.562	0.552	0.046	0.040
*Ia*3̄*d* (γ)	0.571		0.020	
*I*4_1_/*amd*	1.005	1.030	0.000	0.018
*P*6_1_22 (α)	0.828	0.808	0.000	0.000

**Fig. 1:**
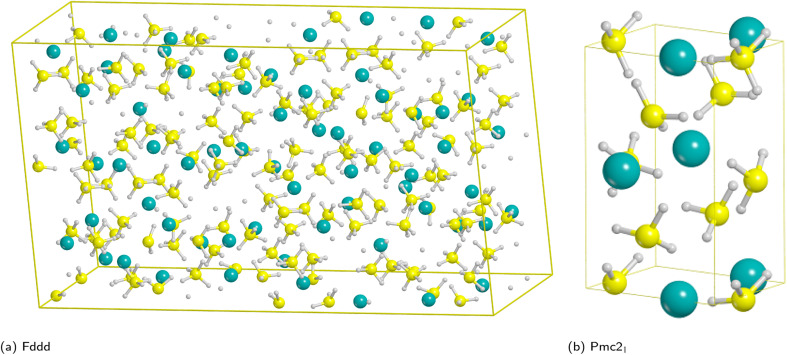
Unit cells of the high temperature *Fddd* Mg(BH_4_)_2_ polymorph (left) and *Pmc*2_1_ model system (right). Hydrogen in white, boron in yellow, magnesium in cyan.

From the experimental viewpoint, the most interesting polymorph is the β phase (*Fddd* space group), because it is stable at high temperature and it decomposes to release hydrogen;^72–75^ however, it contains 704 atoms in the unit cell (Fig. 1a) and, accordingly, the cost of the simulations, especially for surfaces (for which the number of atoms is a multiple of the bulk), would become unaffordable.

Therefore, for the purpose of this work we decided to adopt a simpler model system as similar as possible to the β-phase. We then considered the known experimental polymorphs and theoretically predicted structures of Table 1,^76,77^ for which the relative stability (Δ*E*) with respect to the athermal limit (*T* = 0 K, no zero-point-energy corrections) and the density obtained for each polymorph have been reported.

On the basis of present and previous calculations carried out with the same DFT method the polymorph selected for the surface analysis is the theoretically proposed *Pmc*2_1_ phase (Fig. 1b).^77^

**Fig. 2:**
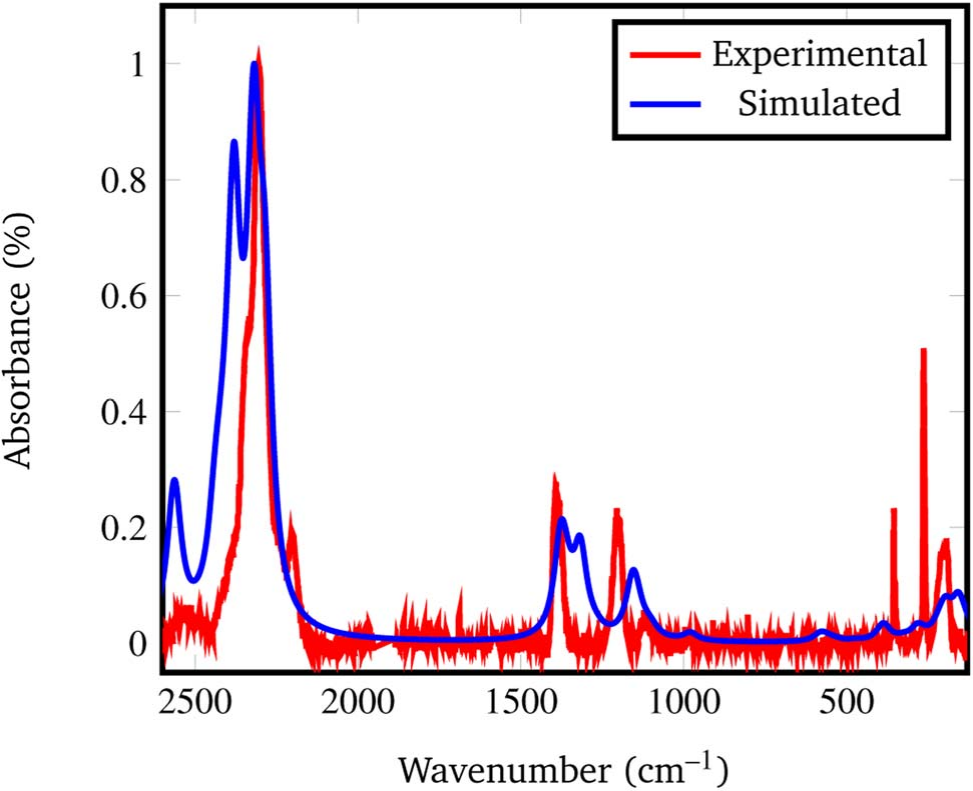
Experimental *vs.* simulated *Pmc*2_1_ Mg(BH_4_)_2_ Raman spectrum. The experimental spectrum was recorded from the *Fddd* β phase, taken and adapted from ref. 78.

In fact, by comparing the densities and the relative energies, it becomes apparent that the *Pmc*2_1_ structure closely mimics the features of the *Fddd* phase. To verify this assumption, in Fig. 2 we report a comparison between the experimental Raman spectrum of the *Fddd* phase^78^*vs.* the simulated one of the *Pmc*2_1_. The match is particularly good, considering that no shift was applied to any frequency, even for the B–H stretching (1800–2500 cm^−1^) and HB̂H bending (1000–1500 cm^−1^) regions, that normally are difficult to reproduce, due to the strong anharmonicity of H-bearing moieties. The position of the peaks is correctly reproduced even in the fingerprint region below 500 cm^−1^. The great advantage of the *Pmc*2_1_ phase is that it has just 22 atoms in the unit cell thus making the calculations faster. Despite this can be considered a too strong simplification, given the complexity of the *Fddd* phase, the evidence above corroborates the hypothesis that the two phases could show a similar behavior toward decomposition. Therefore, in the present study, we adopted the hypothetical *Pmc*2_1_ polymorph (Fig. 1b) to model and understand the properties of Mg(BH_4_)_2_ surfaces and their decomposition reaction along with the role of additives in destabilizing the borohydride.

### 2.2 Methods

The theoretical investigation of Mg(BH_4_)_2_ in its bulk and surface structures was carried out with periodic density functional theory (DFT) calculations by employing mostly the PBE-D*^79^ GGA functional and, to validate some results (*vide infra* for details), the M06-D*^80,81^ hybrid mGGA functional. These methods correspond to the PBE and M06 functionals, respectively, augmented with the Grimme's DFT-D2 (ref. 82 and 83) empirical dispersion correction, as modified for solids by Civalleri *et al.*^84^ The empirical term is based on an atom–atom pairwise C_6,*ij*_/R_*ij*_^6^ contribution (*i.e.* a London-type correction). All calculations were carried out with the *ab initio* CRYSTAL program.^85,86^ Crystalline orbitals are represented as linear combinations of Bloch functions (BF), and are evaluated over a regular three-dimensions mesh of points in reciprocal space. Each BF is built from local atomic orbitals (AO), which are contractions (linear combinations with constant coefficients) of Gaussian-type-functions (GTF) which, in turn, are the product of a Gaussian times a real solid spherical harmonic function. All electron basis sets were used for all the atoms. In particular, we used a 6-311G(d,p) basis set for H, B, F, Cl and Mg and a TZVP for Ni and Cu.^87^ For the numerical integration of the exchange and correlation terms for all calculations we adopted 75 radial points and 947 angular points (XLGRID) in a Lebedev scheme in the region of chemical interest were adopted. The Pack–Monkhorst/Gilat shrinking factors for the reciprocal space were set to 8. The accuracy of the integral calculations was increased by setting the tolerances to 9, 9, 7, 7, 18 for the pure system and 7, 7, 7, 7, 18 for the doped systems. The self-consistent field (SCF) iterative procedure was converged to a tolerance in total energy of Δ*E* = 1 × 10^−7^ a.u., and to accelerate convergence in the self-consistent calculations a modified Broyden's scheme^88^ following the method proposed by Johnson^89^ was adopted; the method was applied after 10 SCF iterations, with 50% of Fock/KS matrices mixing and with the Johnson parameter set to 0.05. As regards the geometry optimization of the slab models, only the atomic positions were optimized while the cell parameters were kept fixed at their optimized values calculated on the bulk structure. Calculations with the additives were carried out on a (2 × 1) supercell model in order to simulate an isolated additive inclusion/substitution and with the unrestricted Kohn–Sham formalism. Vibrational frequencies, referred to the Γ point,^90,91^ were calculated at the optimized geometry by means of mass-weighted Hessian matrix, which is obtained by numerical differentiation of the analytical first derivatives, to be sure that all the structures are minima of the potential energy surface (PES). For MgB_2_ phonon dispersion has been calculated throughout the supercell approach, adopting a (2 × 2 × 2) supercell in order to obtain a cell volume similar to Mg(BH_4_)_2_, thus balancing the number of normal modes between the two different unit cells when calculating decomposition energies. Thermal corrections to the decomposition reactions were carried out at 300, 450, 600 K (see details in Table S1†). Raman intensities were calculated through the CPKS approach (Couple Perturbed Kohn–Sham);^92,93^ to match the experimental spectrum, the temperature and laser wavelength were set to 15 K and 488 nm, respectively.

Regarding the transition state search, a numerical estimation of the initial hessian was requested in order to follow the correct eigenvector and, to this end, the SCF tolerance was increased to Δ*E* = 1 × 10^−11^ a.u. The structures of reactants and products were found by performing a rigid scan along the normal mode of the imaginary frequency of the transition state and optimizing the last point of the scan in both the directions.

## 3 Results and discussion

### 3.1 Surfaces stability

**Fig. 3:**
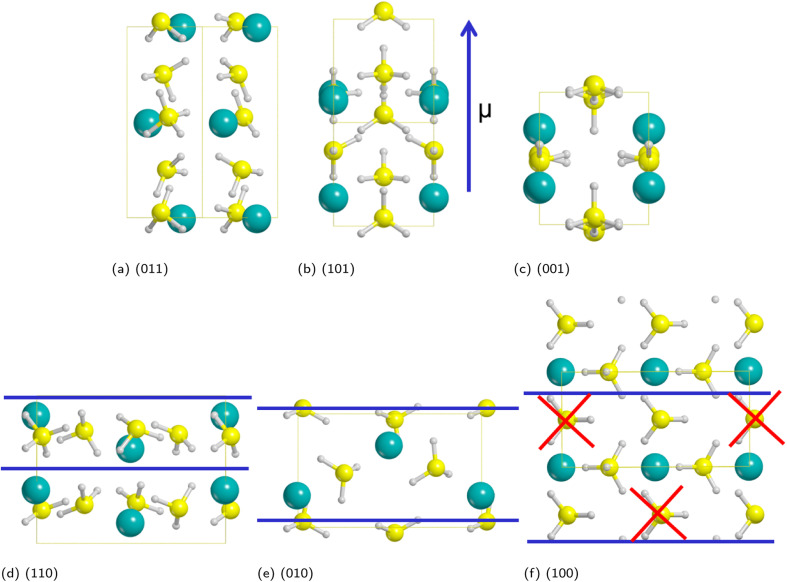
Non-physically (a–c) and physically (d–f) stable surfaces of *Pmc*2_1_ Mg(BH_4_)_2_ phase. The repeat unit needed to create the slab model is highlighted between blue lines. For the (100) face, two BH_4_^−^ units were removed to make the slab stoichiometric. Hydrogen in white, boron in yellow, magnesium in cyan.

As discussed in a previous work on Ca(BH_4_)_2_ surfaces,^49^ it is important to predict which is the most stable surface, because it is the most representative one in experimentally synthesized nanoparticles and, accordingly, the one where the inclusion of the additive occurs with the highest probability. To this purpose we investigated several low-index surfaces, namely: (100), (010), (001), (110), (101) and (011). We adopted a slab model approach (*i.e.* a 2D thin film of a given thickness) that was successfully applied to model the Ca(BH_4_)_2_ surfaces. Here, we briefly recall that a slab model, to be physically stable, must be comprised of stoichiometric (*i.e.* neutral) and non-polar (*i.e.* no net dipole moment perpendicular to the slab) repeat units (RU). Accordingly, three out of them (*i.e.* (001), (101) and (011)) are not physically stable, as shown in Fig. 3. The remaining faces (*i.e.* (110), (010) and (100)) are stoichiometric and non-polar. The repeat units used to create the slab model are reported in Fig. 3. For the (100) surface, a structural rearrangement of the repeat unit is needed to make it stoichiometric. Specifically, two BH_4_^−^ units were removed. Slab models of different thickness from 1-RU up to 7-RU were then investigated to identify the minimum thickness of the slab that leads to converged properties. To this purpose, the surface formation energy (*E*_s_) was used as the reference property and it was computed for all the optimized slabs of the (110), (010) and (100) surfaces according to the following equation:1
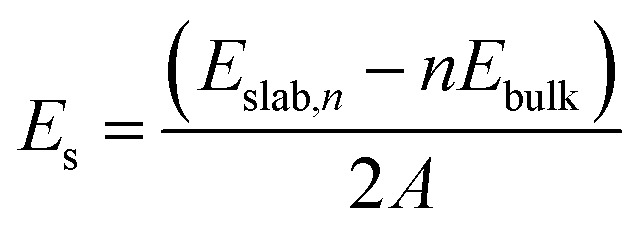
where *E*_slab,*n*_ is the total energy of the slab comprised of *n* RUs, *E*_bulk_ is the total energy of the RU as in the bulk, *n* is the number of RUs and *A* is the area of the 2D unit cell.

**Fig. 4:**
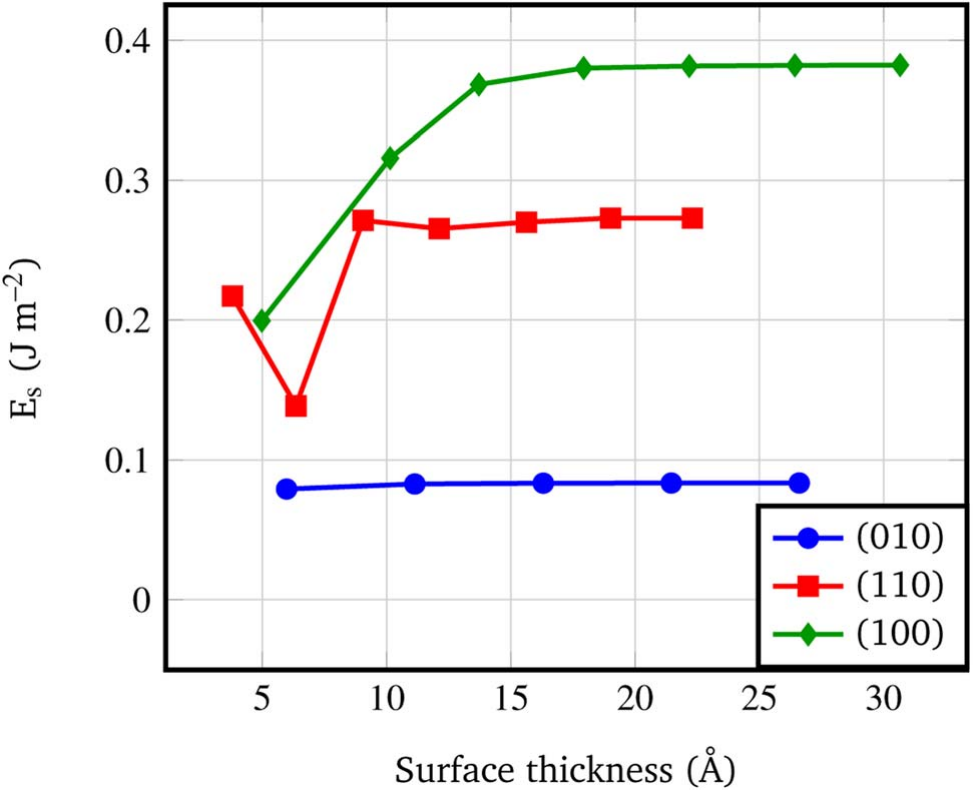
Surface formation energy (J m^−2^) *vs.* slab thickness. The surface thickness corresponds to slab models ranging from 1 to 7 repeat units.

Fig. 4 shows that the *E*_s_ converges to a constant value of about 0.08 J m^−2^ (4-RU), 0.27 J m^−2^ (4-RU) and 0.38 J m^−2^ (2-RU) for (010), (110) and (100) faces, respectively. Overall, the convergence is reached for a slab thickness of about 15 Å (see Fig. 4). It turns out that the relative stability of the different faces is: (010) > (110) > (100), with the (010) surface being significantly more stable than the other two faces.

### 3.2 Nanosizing effects

An important byproduct of the study of the relative stability of the different surfaces of *Pmc*2_1_ Mg(BH_4_)_2_ is the possibility to model nanosizing effects on the decomposition of Mg(BH_4_)_2_. Indeed, one can refer to the slab model as a thin film of a given thickness and consider the decomposition of films of decreasing thickness. In this study, we considered the following decomposition reaction:^94^

**Fig. 5:**
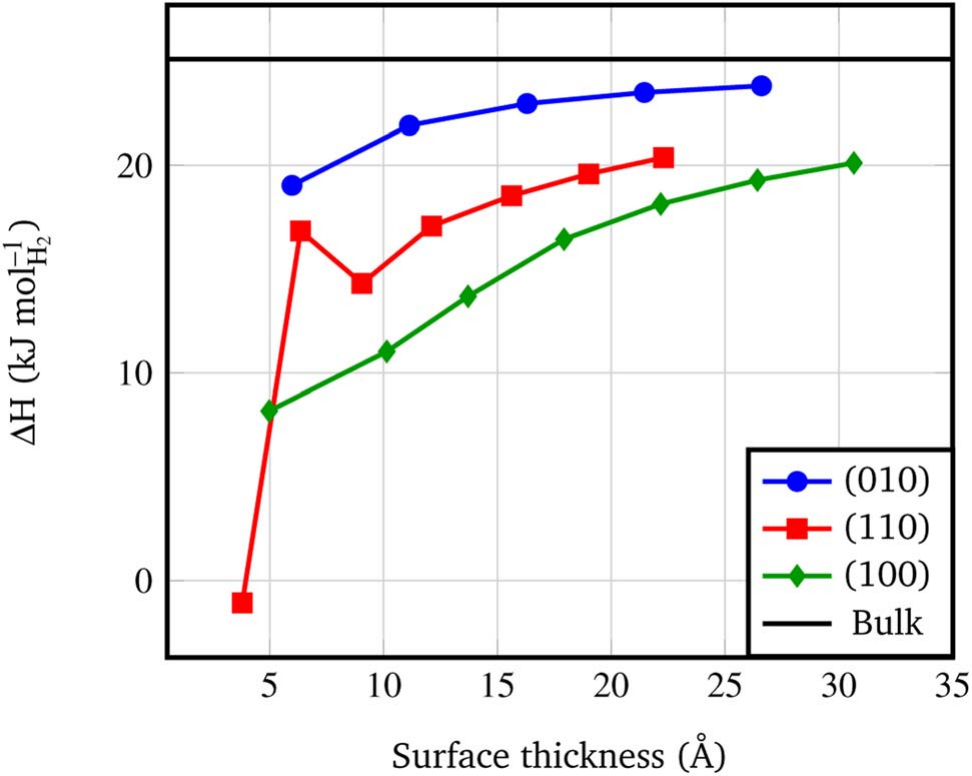
Surface decomposition enthalpies (kJ mol_H_2__^−1^, calculated at 450 K) *vs.* slab thickness. The surface thickness corresponds to slab models ranging from 1 to 7 repeat units. The black line corresponds to the decomposition energy of the *Pmc*2_1_ Mg(BH4)_2_ bulk structure, according to the following reaction: Mg(BH_4_)_2_ → MgB_2_ + 4H_2_.

2 Mg(BH_4_)_2(s)_ → MgB_2(s)_ + 4H_2(g)_

in which, along with molecular hydrogen, solid magnesium boride is formed. Other possible decomposition reaction paths have been proposed in the literature, but currently there is a general consensus that MgB_2_ is formed as main product.^94^ We also considered the decomposition reaction to solid magnesium hydride and α-boron but the corresponding computed data are available as ESI in Fig. S1.† Results for reaction above are shown in Fig. 5 and compared to the decomposition enthalpy calculated on the bulk structure at 450 K. It can be clearly seen that the decomposition enthalpy drops down when the thickness of the thin film decreases. This confirms that nanosizing can lead to a remarkable reduction of the decomposition enthalpy when the slab thickness is below 2 nm. As expected, effects are more pronounced for the less stable surfaces with a decrease in the decomposition enthalpy between 10 and 20 kJ mol_H_2__^−1^. When considering that nanosized Mg(BH_4_)_2_ particles expose different surfaces at the environment, a net reduction of the decomposition enthalpy of 15 kJ mol^−1^ is predicted to occur. It is worthy to note that the full pathway accounting for the complete decomposition reaction of Mg(BH_4_)_2_ is a quite complex task to be simulated, and the possible intermediates are many, as studied in several previous computational papers.^94–97^ Here we prefer to focus on the final step of the decomposition to highlight the differences of using nanosized films instead of the bulk structure.

### 3.3 The role of additives

**Fig. 6:**
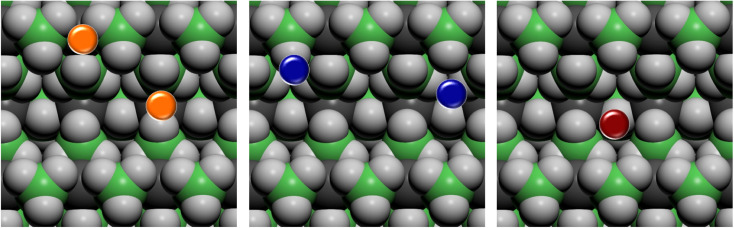
Top view of possible doping sites on the *Pmc*2_1_ Mg(BH_4_)_2_ (010) surface for the different additive species. Substitutional defective site (Ni^2+^) in orange, interstitial defective site (Ni^0^) in blue, doping site of nickel and copper halides (NiF_2_, NiCl_2_ and CuF_2_) in red. Hydrogen in light grey, boron in green, magnesium in dark grey.

On the basis of the results of the surface stability, we simulated the inclusion of different additives on top of the *Pmc*2_1_ Mg(BH_4_)_2_ (010) surface model. In particular, according to the recent study carried out by Hauback and coworkers^69^ on the role of Ni-based additives in the decomposition of Mg(BH_4_)_2_, three different kinds of additive species were explored, namely: the substitutional defect (Ni^2+^), the interstitial defect (Ni^0^), and the doping with nickel halides (*i.e.* NiF_2_ and NiCl_2_). Along with NiF_2_, we also modeled the inclusion of CuF_2_ on the surface. This allowed us to compare the results for two transition metals and to evaluate their performance toward the decomposition process. For the calculations, a slab model of about 11 Å (2 RU) of thickness was adopted and a 2 × 1 supercell was employed to reduce the density of additives on the surface. According to the structure of the *Pmc*2_1_ Mg(BH_4_)_2_ (010) surface different sites for the Ni-based additives can be investigated. Fig. 6 shows the possible locations of the doping sites for the different additive species studied in the present work. For the substitutional and the interstitial additive two possible sites were explored around the outermost and the innermost Mg ions, respectively, while for the metal halides a single site was modelled, due to the larger size of the additive, that fit the cavity on the surface.

**Fig. 7:**
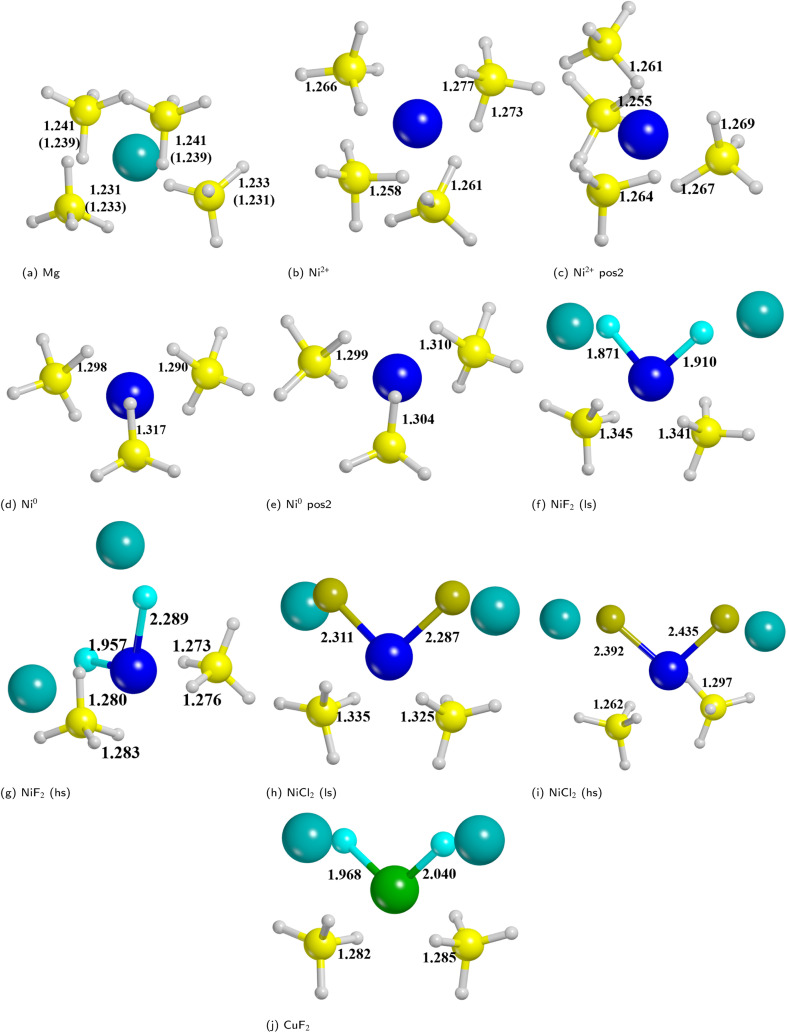
Metal ion/atom/halide with the surrounding borohydrides as extracted from the optimized slab models for bare and doped surfaces. (a) Bare surface (the numbers in parenthesis refers to deeper borohydrides); (b) substitutional defect of outermost Mg; (c) substitutional defect of innermost Mg; (d) interstitial defect in the middle of outermost BH_4_^−^; (e) interstitial defect in the middle of innermost BH_4_^−^; (f) NiF_2_ additive (low spin); (g) NiF_2_ additive (high spin); (h) NiCl_2_ additive (low spin); (i) NiCl_2_ additive (high spin); (j) CuF_2_ additive. Some relevant B–H and M–X distances (in Å) are reported. Hydrogen in white, boron in yellow, magnesium in cyan, nickel in blue, copper in green.

We analyzed the results on the basis of the structural deformation that the additive produces to the surrounding borohydride ions with respect to the bare material. As a general evidence, the presence of the additive in the structure leads to a remarkable rearrangement of its first coordination sphere. The closest BH_4_^−^ ion, indeed, appears very distorted with respect to the pure system. The deformation of the B–H bond lengths can then be considered as an indicator of the propensity of the borohydride to decompose. It turns out that the longer the B–H bond distance (*i.e.* the weaker the B–H bond), the easier the decomposition. A comparison between the optimized structures without and with the Ni based additives and CuF_2_ is reported in Fig. 7. For sake of simplicity, the metal atom/ion/halide with the surrounding borohydrides is shown as extracted from the optimized slab models.

As expected, the geometry around the substitutional defective site is perturbed by the substitution with Ni^2+^, but it maintains a typical tetrahedral coordination. For the interstitial defective site, instead, the Ni^0^ atom tends to rearrange the BH_4_^−^ groups in a trigonal-planar coordination with three borohydrides in closer contact with the metal center. In the case of the metal halides additives, both Ni and Cu are tetra-coordinated with two halide atoms and two BH_4_^−^ groups.

**Fig. 8:**
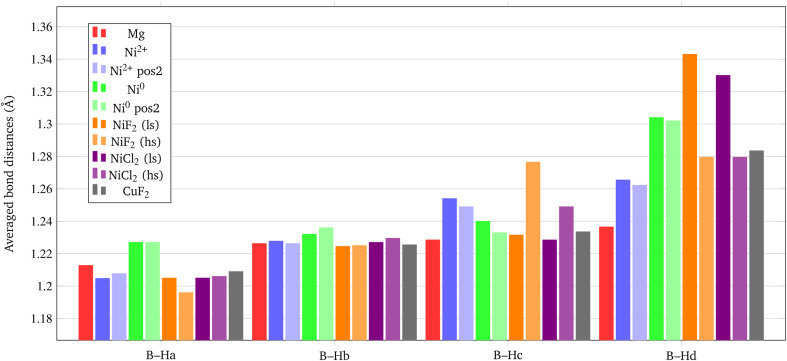
Average B–H bond distances (in Å) for the BH_4_^−^ groups closest to the metal center.

The structural deformation of the BH_4_^−^ groups around the metal can be clearly seen from the B–H bond distances as reported in Fig. 7. For all additive species, an elongation of the B–H bonds is observed with respect to the non-doped surface. In the chart of Fig. 8, the B–H bond distances (in Å) for the four nearest neighbouring BH_4_^−^ groups of the metal are shown. On average, the largest elongation is predicted for atomic Ni as an interstitial site. In the other cases, one BH_4_^−^ groups out of four is definitely more perturbed by the presence of the additive. The effect of the metal on the B–H bond is particularly relevant for both NiF_2_ and NiCl_2_. Interestingly, CuF_2_ is not as effective as Ni-based additives in perturbing the BH_4_^−^ groups. Therefore, it is expected that Ni would outperform Cu in favouring the decomposition of Mg(BH_4_)_2_.

**Fig. 9:**
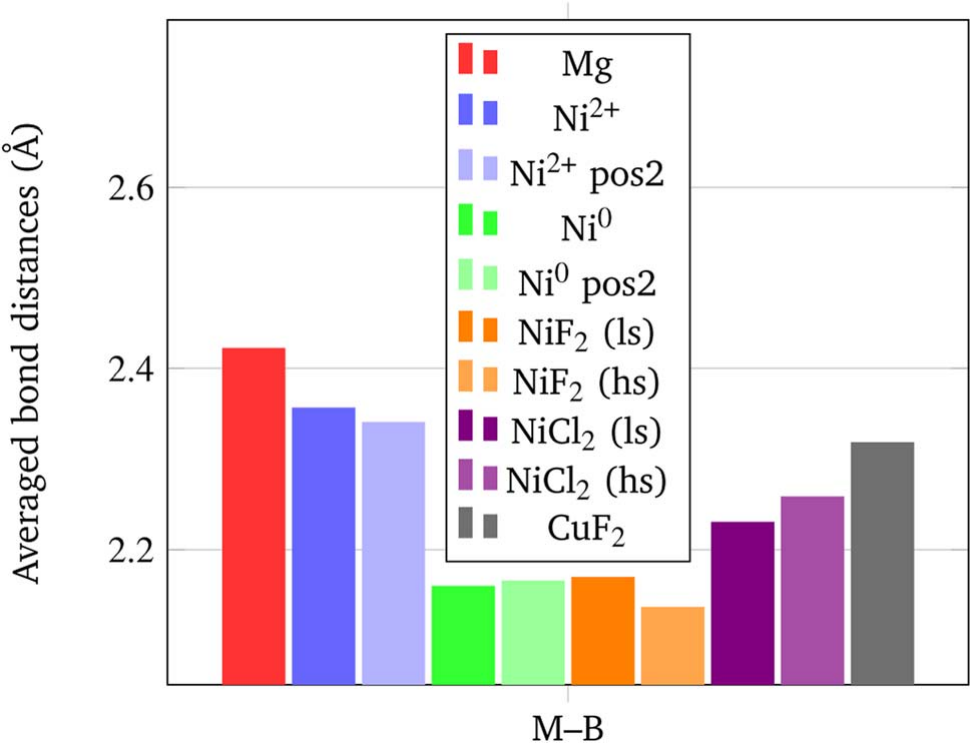
Average M–B bond distances (in Å) for the BH_4_^−^ groups closest to the metal center.

In addition, it is important to point out that also the Ni–B distance can be used as a further indicator of the increased propensity of the borohydride toward dissociation. In fact, it has been shown by Saldan *et al.*^69^ that the decomposition of Mg(BH_4_)_2_ always occurs concurrently with the formation of the metal boride. Therefore, a shorter Ni–B distance can be interpreted as an increased tendency to the decomposition of the BH_4_^−^ groups. The mechanism of Mg(BH_4_)_2_ involve a redox reaction where the H is oxidized from −1 in the [BH_4_]^−^ group to its native state in the H_2_ molecule, and the boron is reduced from +3 to −1 in the metal boride. As clearly seen in Fig. 9, Ni^0^ in the interstitial site leads to a remarkable shortening of the mean Ni–B distance in the borohydride, followed by Ni and Cu halides. Details on structural features are available in Fig. S1–S18 and Tables S1–S9.†

On the latter system (*i.e.* NiF_2_), which seems to be the most promising one in promoting the decomposition of Mg(BH_4_)_2_, we also improved the level of theory to have a better description of the nickel spin state and validate the PBE-D* results. Therefore, we relied on the more accurate rung of the Jacob's ladder of DF approximations by using a hydrid functional, the M06-D*, which has been shown to be particularly good in describing transition metals and, moreover, because the inclusion of a 27% of the exact Hartree–Fock exchange allows a more correct localization of unpaired electrons. According to PBE-D* results, we found a more stable structure for the Ni in high spin state (see Fig. S15, S16 and Table S9†) instead of the low spin (see Fig. S13, S14 and Table S8†), with a difference of Δ*E* = 34.22 kJ mol^−1^ and Δ*H* = 35.14 kJ mol^−1^. M06-D* confirms this result from a qualitative point of view, but it further stabilizes the high spin state solution with a Δ*E* of 79.33 kJ mol^−1^. Differences in the geometries with the two levels of theory are negligible on B–H distances, even if a systematic increasing of the bond distances of PBE-D* with respect to M06-D* is predicted. In contrast, M06-D* tends to elongate the Ni–B distance with respect to PBE-D*, and in this case the difference in the two geometries is larger, thus indicating that a proper description of the metal where the spin is localized is crucial. Details on the comparison between PBE and M06 are in Fig. S19–S21.†

### 3.4 NiF_2_ and CuF_2_ additives reactivity

In this section we study the reactivity of NiF_2_, the most promising additive according to the results provided in the previous section, as compared to CuF_2_ to confirm the stronger catalytic effect of Ni. Since F atom shows a rather small ionic radius with respect to other halides, it is well known that it tends to substitute H atoms of BH_4_^−^ to form BH_(4−*n*)_F_*n*_^−^ groups.^98^ Then we explored a hypothetical reaction pathway from NiF_2_ through Ni–H_2_, *i.e.* with H atoms chemisorbed on the metal centre, to Ni⋯H_2_ where molecular hydrogen is physisorbed on the surface, until the complete H_2_ desorption (Ni + H_2_). The same strategy was also adopted for CuF_2_ additive.

#### 3.4.1 NiF_2_

From the optimized structure of NiF_2_ in the low spin state on the Mg(BH_4_)_2_ (010) surface, which will be the reference structure, we investigated two possible substitution sites for F atom on the neighbouring BH_4_^−^ groups as depicted in Fig. S23.† Accordingly, from the reactant, two intermediate structures were modelled in which one H atom is substituted by one F atom, thus forming Ni–I1 (Δ*H* = −27.2 kJ mol^−1^) and Ni–I2 (Δ*H* = 48.0 kJ mol^−1^) (see Fig. S23c and d†). From these intermediates (hereafter referred to as Ni–I1 and Ni–I2) the two corresponding products (denoted as Ni–P1 and Ni–P2) are formed, Ni–P1 (104.6 kJ mol^−1^) and Ni–P2 (76.9 kJ mol^−1^), where two H atoms are transferred on the metal centre and F atoms on two different BH_4_^−^ groups (see Fig. S23e and f†).

From the intermediate Ni–I2 (see Fig. S24d†), a different product with respect to the above-mentioned was modelled because of the formation of a BH_2_F group and its proximity with the other F atom bound to the nickel. We then built up another product in which two F atoms substitute the same BH_4_^−^ to form a BH_2_F_2_^−^ group, as shown in Fig. S24g† (Ni–P3, Δ*H* = −68.1 kJ mol^−1^). This structure is more stable than Ni–P1 and Ni–P2, indicating that multiple substitutions on the same BH_4_^−^ group lead to a more favorable product. This is not unexpected because it has already been shown in the case of F^−^ substitutions in LiBH_4_.^98^

Interestingly, the Ni–P3 product also present an incipient H_2_ molecule chemisorbed on Ni, which is expected to be more prone to dissociate and release molecular hydrogen than Ni–P1 and Ni–P2. Therefore, we further explored the reaction path by desorbing the quasi-H_2_ molecule. This allowed us to evaluate the cost of the reaction also from a kinetic point of view by calculating the reaction barrier. Thus, we identified the transition state (Ni-TS) with an activation barrier equal to 36.5 kJ mol^−1^ with respect to Ni–P3, and, from the Intrinsic Reaction Coordinate (IRC) calculation performed over the transition state, we landed to the product with a formal H_2_ molecule physisorbed on the surface (Ni–P3–H_2_, −62.3 kJ mol^−1^ see Fig. S24i†), *i.e.* not chemically bound to the Ni. Finally, we optimized the final product with the H_2_ released, *i.e.* not interacting at all with the surface (Ni–P3 + H_2_ −58.4 kJ mol^−1^, see Fig. S24j†).

#### 3.4.2 CuF_2_

In Fig. S25† the same reaction pathway, but with the CuF_2_ as additive is reported. In this case, only the double F substitution on the same BH_4_^−^, *i.e.* the most promising reaction pathway for Ni, was modelled. As it can be seen in Fig. S25c,† despite the stabilization due to the ionic interactions of F atoms with Mg atoms, the product Cu–P is endergonic (18.6 kJ mol^−1^), *i.e.* not as stable as Ni–P3. Notably, in this case, there is no evidence of the formation of the incipient H_2_ molecule. In fact, the structure keeps a pseudo-square planar conformation with a distance between the two H atoms of about 2 Å. This geometrical arrangement suggests a more difficult H_2_ release with respect to the NiF_2_ additive.

The IRC calculation leads to a product (Cu–P–H_2_ −4.2 kJ mol^−1^, see Fig. S25e†) in which the metal adopts a different coordination sphere with respect to the previous reactant and intermediates, due to the changing of the oxidation number of Cu. As for the NiF_2_ doped system after the H_2_ release, one can observe a strong rearrangement of the structure. Curiously, we obtained a porous structure (see Fig. S24d–f†), as one can see from the cylindrical channels of the slab structure, which somehow reminds the *Fddd* polymorph.

#### 3.4.3 Comparison between NiF_2_ and CuF_2_

**Fig. 10:**
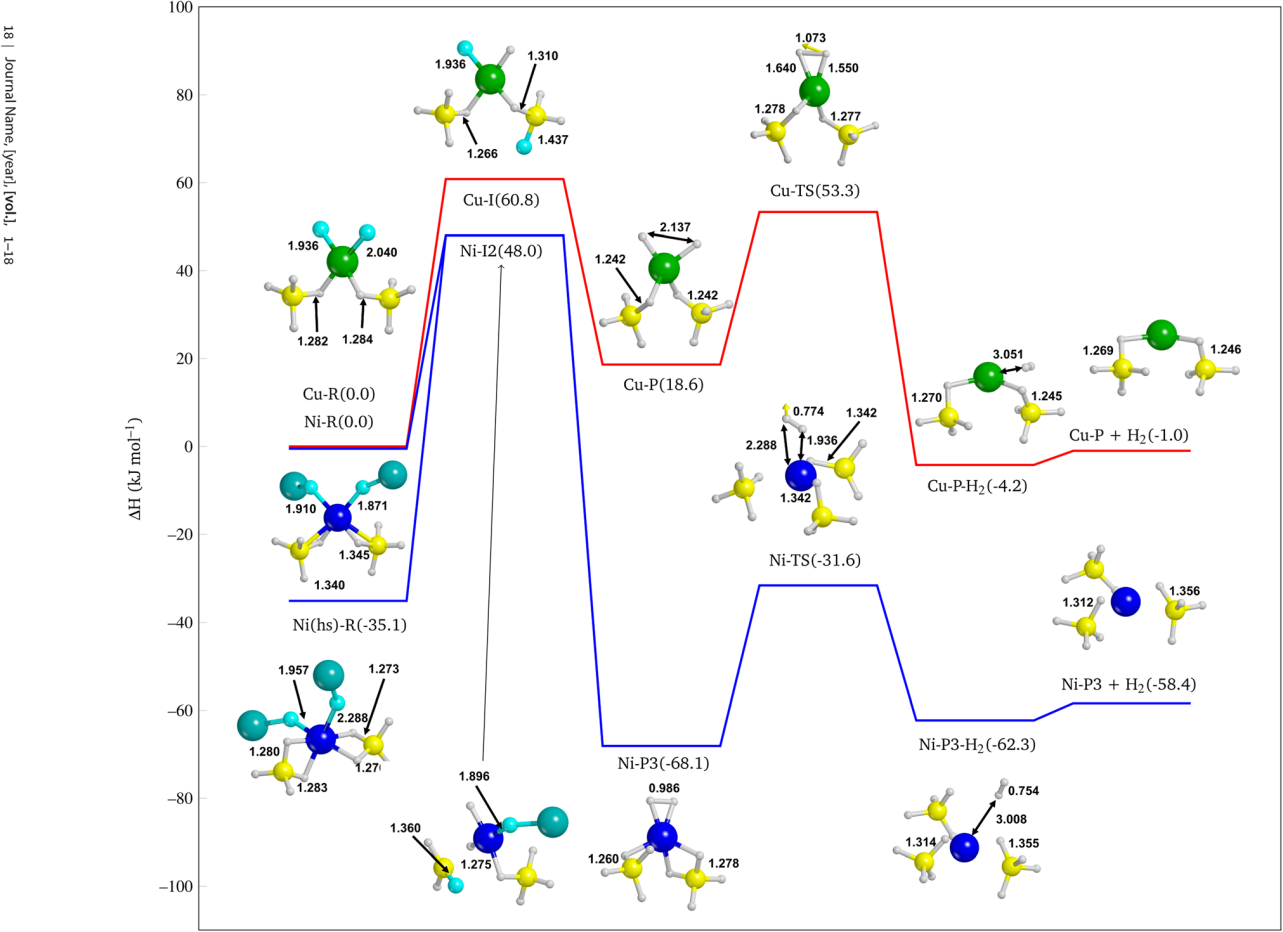
Reaction pathway for the NiF_2_ (blue line) and CuF_2_ (red line) doped systems. Details cut from the periodic structures. Bond distances are in Å. Energy values are in kJ mol^−1^. Boron in yellow, magnesium in dark cyan, nickel in blue, copper in green, fluorine in cyan and hydrogen in white.

In Fig. 10, the enthalpy of the reaction pathway from reagents to products is graphically sketched for both NiF_2_ and for CuF_2_ additives. While the formation of the intermediate (both for NiF_2_ and for CuF_2_) is endothermic, Ni–P3 is favoured because of the stability of the chemisorbed H_2_ molecule, as already mentioned, but also because of the combination of the stabilization of the BH_2_F_2_^−^ group and the attractive ionic interactions between F and Mg. On the contrary for Cu–P, there is no additional stabilizing effect of the H_2_ incipient molecule, thus resulting in an endothermic Cu–P product (18.6 kJ mol^−1^). The reason is that Ni d orbitals can accept part of the electron density of the H–H bond to establish two Ni–H bonds; this results in a typical H–H distance of chemisorbed hydrogen molecule (from the typical 0.75–0.76 Å to 0.99 Å) as a result of the electron density depletion. It turns out that the H_2_ release is more favorable, and this is confirmed by the transition state structure, which is not so high in energy with respect to the Ni–P3 structure (36.5 kJ mol^−1^). In the step between chemisorbed and physisorbed H_2_, the redox reaction occurs, in which the Ni and Cu atoms are reduced from ionic (+2) to atomic (0), and two H atoms are oxidized (from −1 to 0). Interestingly, during this process Ni goes deep into the structure coordinating three BH_4_^−^ groups, thus giving a very similar structure to the Ni(0) inclusion (see Fig. 7d and e).

## 4 Conclusions

In this work we modelled nanosizing effects and the role of additives on the decomposition of Mg(BH_4_)_2_ through quantum mechanical calculations. On the basis of present results we can draw the following conclusions:

• The Mg(BH_4_)_2_*Pmc*2_1_ phase, which does not exist in nature, appears to be a physically reasonable model system of the Mg(BH_4_)_2_ β-phase in terms of density, relative stability, and vibrational features.

• At nanoscale, thin films present a lower decomposition enthalpy with respect to the bulk up to 20 kJ mol^−1^. Not surprisingly, according to the surface stability, faces exposed at the surface show different decomposition enthalpy: the lower the stability of the surface, the lower the decomposition enthalpy.

• Among the examined additives, the best candidate to promote Mg(BH_4_)_2_ decomposition is NiF_2_, whereas Cu is not so effective. This can be simply deduced by the elongation of some B–H bonds and, at the same time, by the decreasing of M–B bond distance. These two indicators can be interpreted as the tendency of the [BH_4_]^−^ to dissociate, thus forming H_2_ on one side and Ni–B species on the other side. Although the effectiveness of Ni agrees with the findings of ref. 69, our results are to be considered from a qualitative viewpoint, as it is not possible to compare them with experimental re-hydrogenation temperature and pressure at this stage.

• Finally, we confirmed the evidence above by investigating the reaction pathway for the H_2_ release from the NiF_2_ and CuF_2_ doped Mg(BH_4_)_2_ (010) surfaces. The most important reactive step that determines the higher efficiency of Ni with respect to Cu in catalysing the reaction is the formation of an incipient hydrogen molecule chemisorbed on the metal. For Ni, this step is exothermic, while for Cu is endothermic of about 20 kJ mol^−1^. Indeed, the H–H bond length in Ni–P3 is very closed to molecular H_2_ (*i.e.* 0.99 Å *vs.* 0.76 Å) thus promoting the reaction towards the final product. From Fig. 10 it is also clear the major catalytic effect of the NiF_2_ doped system, as all its structures are more stable than the CuF_2_ doped one.

In conclusion, the present theoretical work corroborates the evidence that nanostructured metal borohydrides show advantages for energy storage applications compared to their bulk counterparts and that the role of additives as the doping with transition metals (*e.g.* Ni) can further facilitate the release of H_2_.

## Conflicts of interest

There are no conflicts to declare.

## Supplementary Material

RA-014-D3RA08710G-s001
