# Supplementary material for: Theoretical prediction of nanosizing effects and role of additives in the decomposition of Mg(BH4)2
Source: RSC Adv. 2024 Feb 20;14(9):6398–409. doi: 10.1039/d3ra08710g (PMC10877581; doi:10.1039/d3ra08710g)
Supplement: RA-014-D3RA08710G-s001 [file RA-014-D3RA08710G-s001.pdf]

Electronic Supplementary Material (ESI) for RSC Advances.
This journal is © The Royal Society of Chemistry 2024

Theoretical prediction of nanosizing effects and role of additives in the decomposition of $Mg(BH_4)_2$

Supplementary information

Stefano Pantaleone, Elisa Albanese, Lorenzo Donà, Marta Corno, Marcello Baricco, and Bartolomeo Civalleri*

Dipartimento di Chimica e Centro Interdipartimentale NIS, Università di Torino, Via P. Giuria 7 10125 Torino (Italy)

E-mail: bartolomeo.civalleri@unito.it

Decomposition reaction: $Mg(BH_4)_2 \longrightarrow MgBH_2 + 2B + 3H_2$

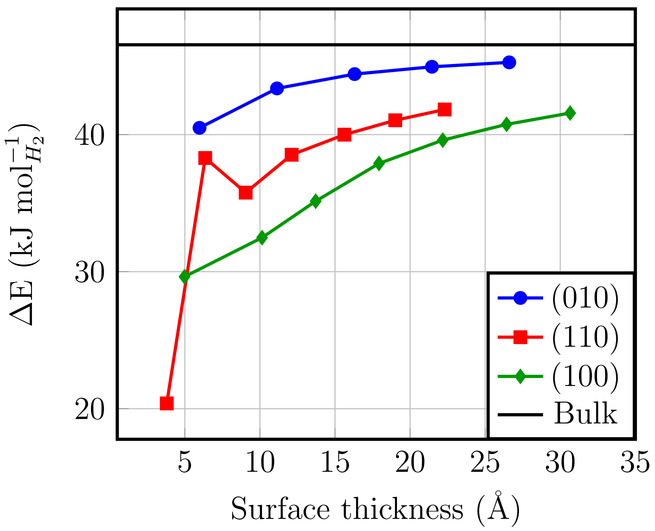

Figure S1: Surface decomposition energies (kJ $mol^{-1}_{H_2}$) *vs* slab thickness. The surface thickness corresponds to slab models ranging from 1 to 7 repeat units.

Decomposition reaction: $Mg(BH_4)_2 \longrightarrow MgB_2 + 4H_2$

Table S1: Decomposition reaction of $Mg(BH_4)_2 \longrightarrow MgB_2 + 4H_2$. E is the electronic energy, E_0 the electronic energy + zero point energy correction, H the enthalpy calculated at different temperatures. Energies are in kJ/mol.

	E	E_0	H^{300}	H^{450}	H^{600}
ΔE	37.50	14.07	25.10	26.11	26.08

Figures and Tables: details on geometries

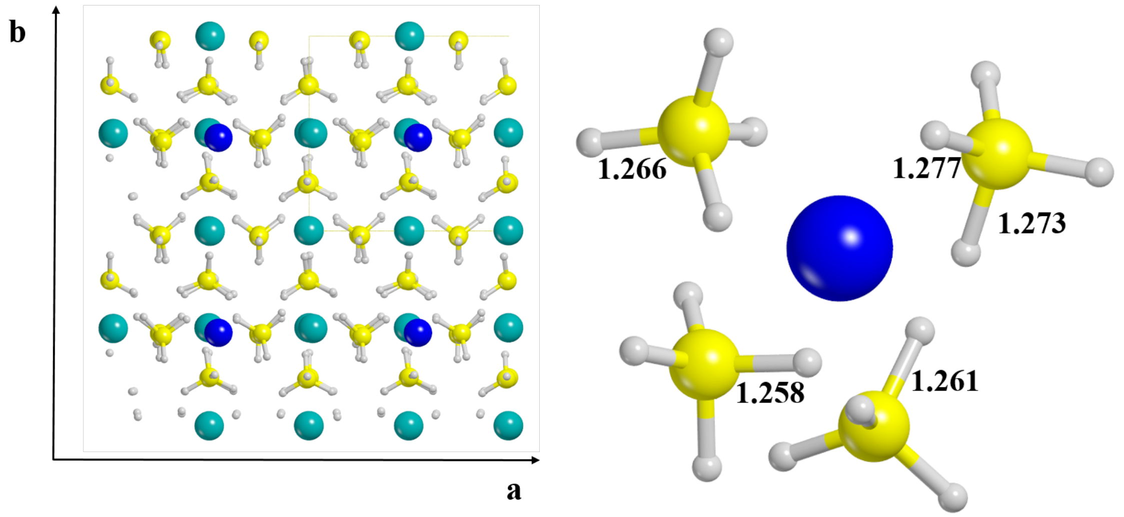

(a) Periodic structure (b) Cluster

Figure S2: Periodic (left) and cluster (right) as extracted from the periodic structure.

Table S2: Bond distances of the system doped with Ni^{2+}.

Bond	Bond distance (Å)			
NiB	2.150	2.160	2.546	2.570
BH1	1.202	1.218	1.203	1.196
BH2	1.232	1.224	1.225	1.230
BH3	1.273	1.257	1.243	1.243
BH4	1.277	1.266	1.258	1.261

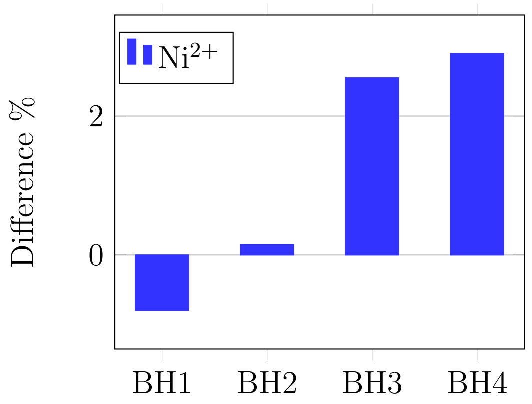

Figure S3: Percentage difference of B–H bond lenghts of the doped system respect to $Mg(BH_4)_2$ pure system.

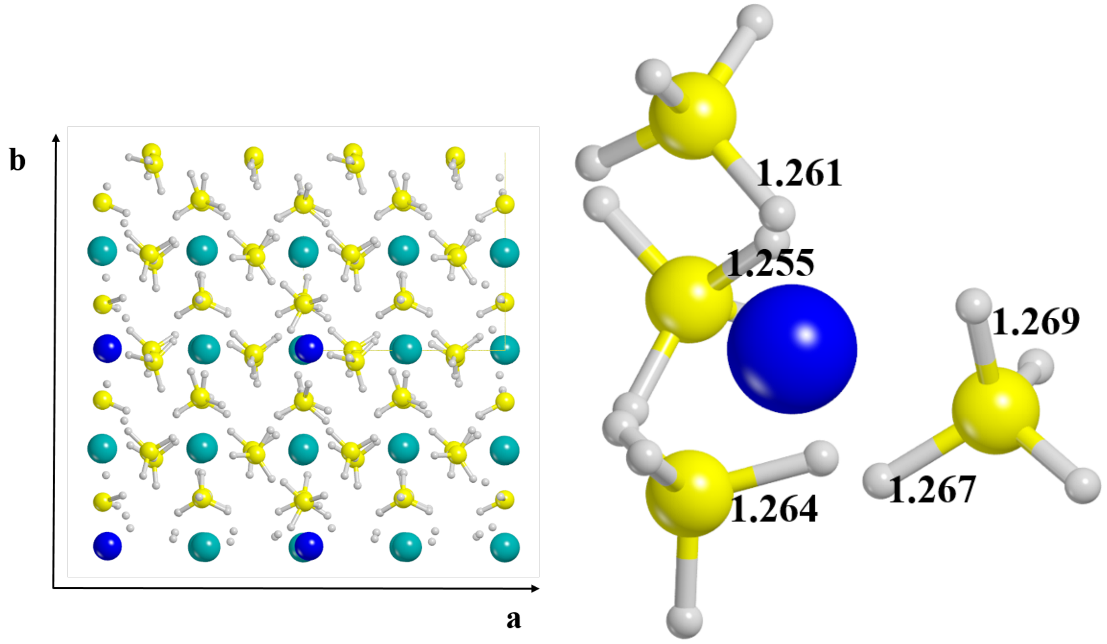

(a) Periodic structure (b) Cluster

Figure S4: Periodic (left) and cluster (right) as extracted from the periodic structure.

Bond	Bond distance ($\AA$)			
NiB	2.167	2.167	2.504	2.523
BH1	1.217	1.200	1.202	1.212
BH2	1.220	1.234	1.226	1.225
BH3	1.256	1.267	1.238	1.235
BH4	1.264	1.269	1.261	1.255

Table S3: Bond distances of the system doped with Ni^{2+} (position 2).

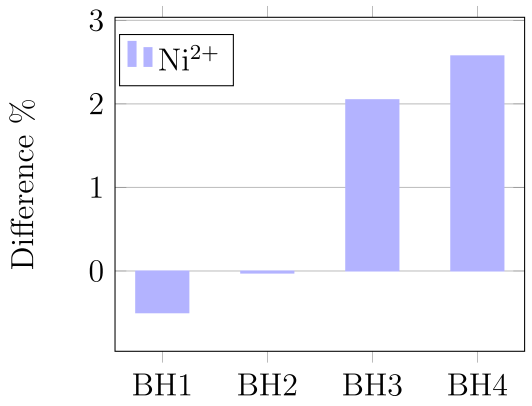

Figure S5: Percentage difference of B–H bond lenghts of the doped system (position 2) respect to $Mg(BH_4)_2$ pure system.

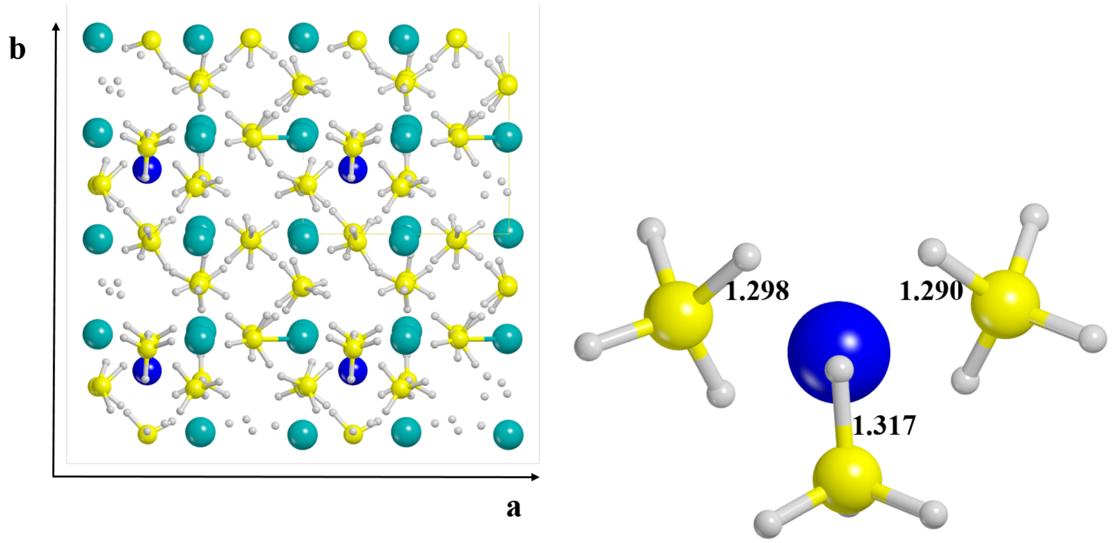

(a) Periodic structure (b) Cluster

Figure S6: Periodic (left) and cluster (right) as extracted from the periodic structure.

Bond	Bond distance (Å)		
NiB	2.089	2.182	2.207
BH1	1.229	1.225	1.228
BH2	1.233	1.236	1.230
BH3	1.258	1.239	1.235
BH4	1.317	1.298	1.290

Table S4: Bond distances of the system doped with Ni^0.

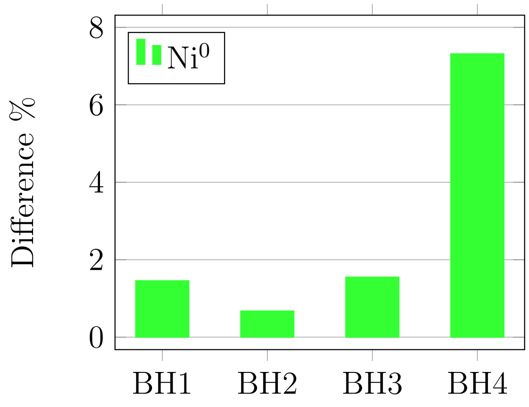

Figure S7: Percentage difference of B–H bond lenghts of the doped system respect to $Mg(BH_4)_2$ pure system.

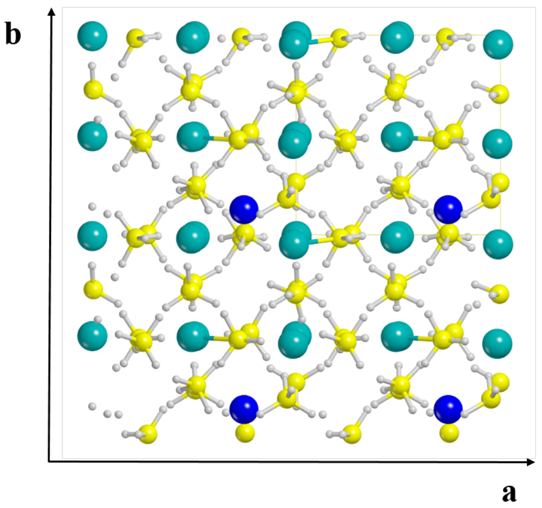

(a) Periodic structure

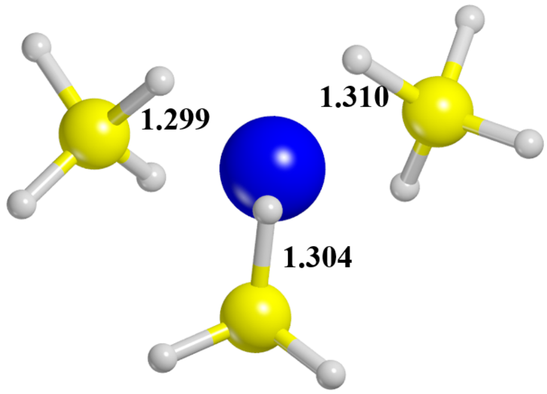

(b) Cluster

Figure S8: Periodic (left) and cluster (right) as extracted from the periodic structure.

Bond	Bond distance (Å)		
NiB	2.098	2.148	2.225
BH1	1.225	1.232	1.225
BH2	1.234	1.238	1.225
BH3	1.254	1.240	1.226
BH4	1.304	1.310	1.299

Table S5: Bond distances of the system doped with Ni^0 (position 2).

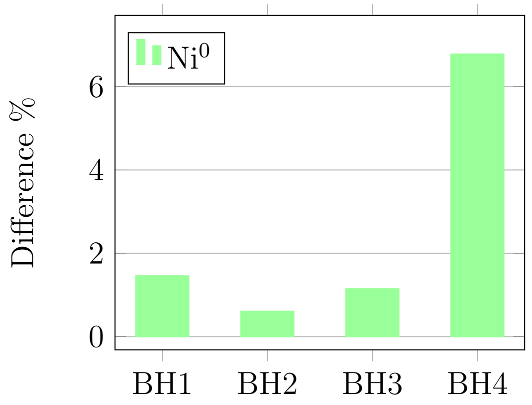

Figure S9: Percentage difference of B–H bond lenghts of the doped system (position 2) respect to $Mg(BH_4)_2$ pure system.

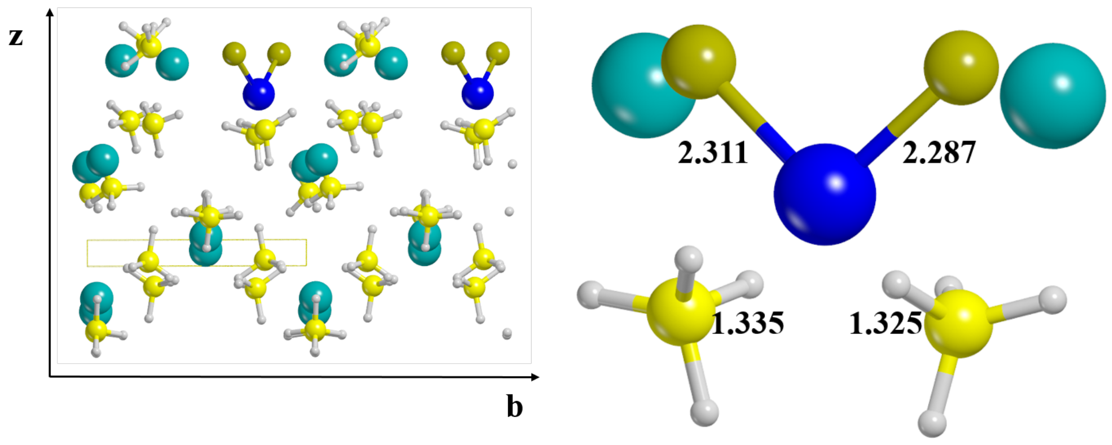

(a) Periodic structure (b) Cluster

Figure S10: Periodic (left) and cluster (right) as extracted from the periodic structure.

Bond	Bond distance ($\AA$)	
NiB	2.228	2.232
NiCl	2.287	2.311
BH1	1.205	1.205
BH2	1.230	1.224
BH3	1.232	1.225
BH4	1.325	1.335

Table S6: Bond distances of the system doped with $NiCl_2$ (low spin).

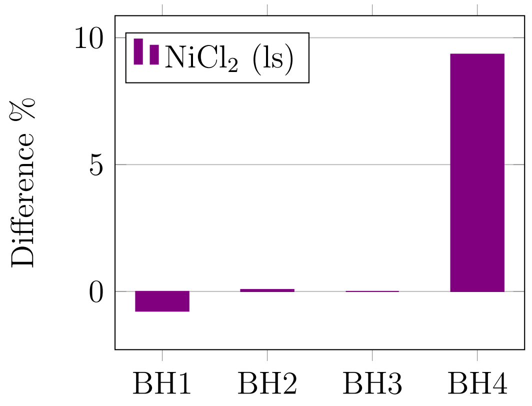

Figure S11: Percentage difference of B–H bond lenghts of the doped system respect to $Mg(BH_4)_2$ pure system.

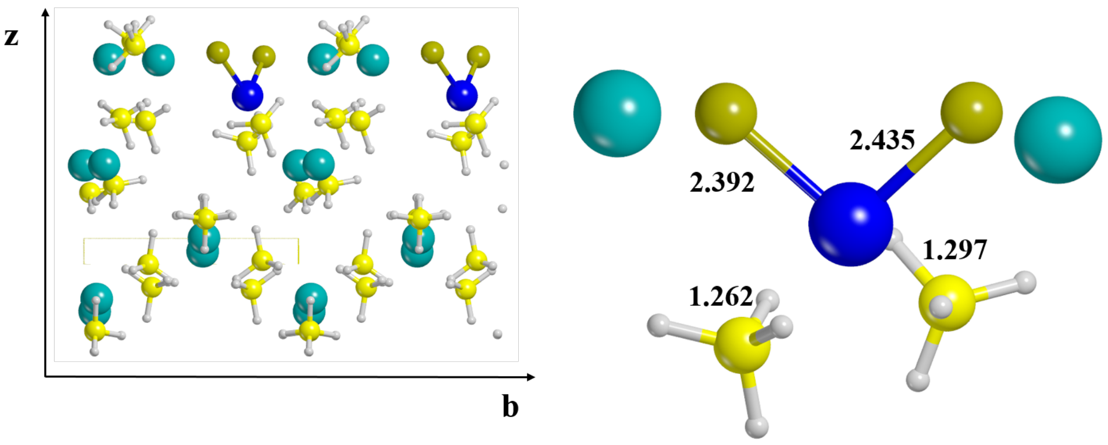

(a) Periodic structure (b) Cluster

Figure S12: Periodic (left) and cluster (right) as extracted from the periodic structure.

Bond	Bond distance (Å)	
NiB	2.113	2.403
NiCl	2.435	2.392
BH1	1.201	1.211
BH2	1.230	1.229
BH3	1.268	1.230
BH4	1.297	1.262

Table S7: Bond distances of the system doped with $NiCl_2$ (high spin).

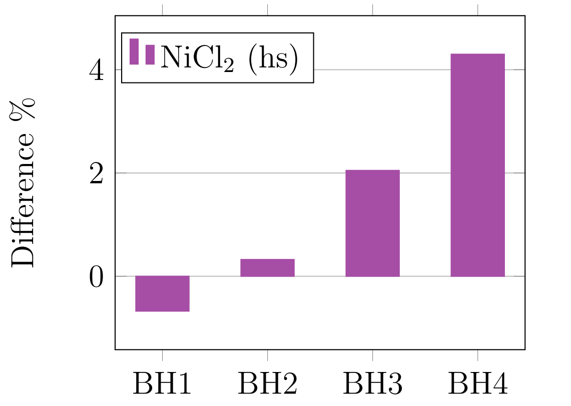

Figure S13: Percentage difference of B–H bond lenghts of the doped system respect to $Mg(BH_4)_2$ pure system.

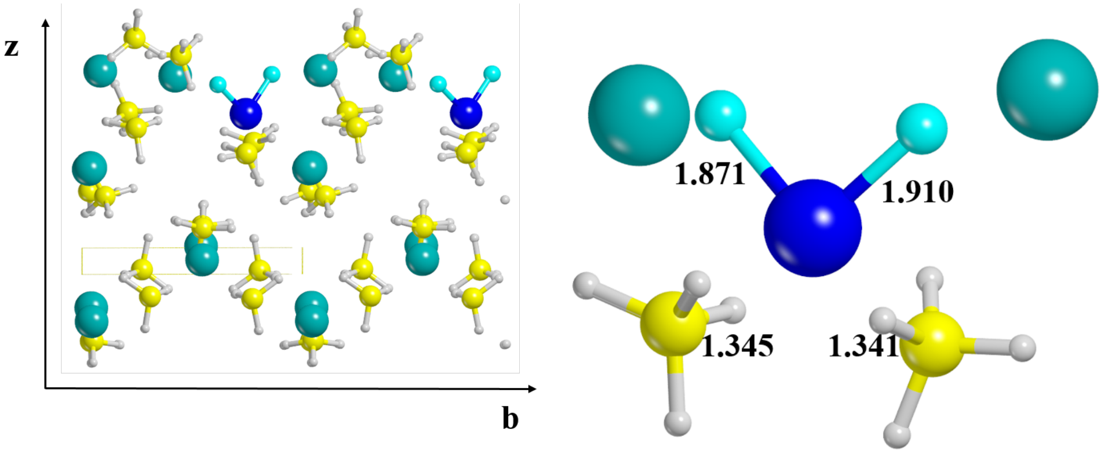

(a) Periodic structure (b) Cluster

Figure S14: Periodic (left) and cluster (right) as extracted from the periodic structure.

Bond	Bond distance ($\AA$)	
NiB	2.187	2.192
NiF	1.910	1.871
BH1	1.211	1.208
BH2	1.232	1.227
BH3	1.234	1.229
BH4	1.352	1.356

Table S8: Bond distances of the system doped with NiF_2 (low spin).

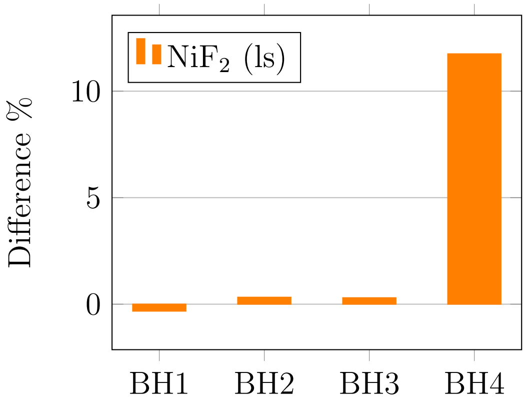

Figure S15: Percentage difference of B–H bond lenghts of the doped system respect to $Mg(BH_4)_2$ pure system.

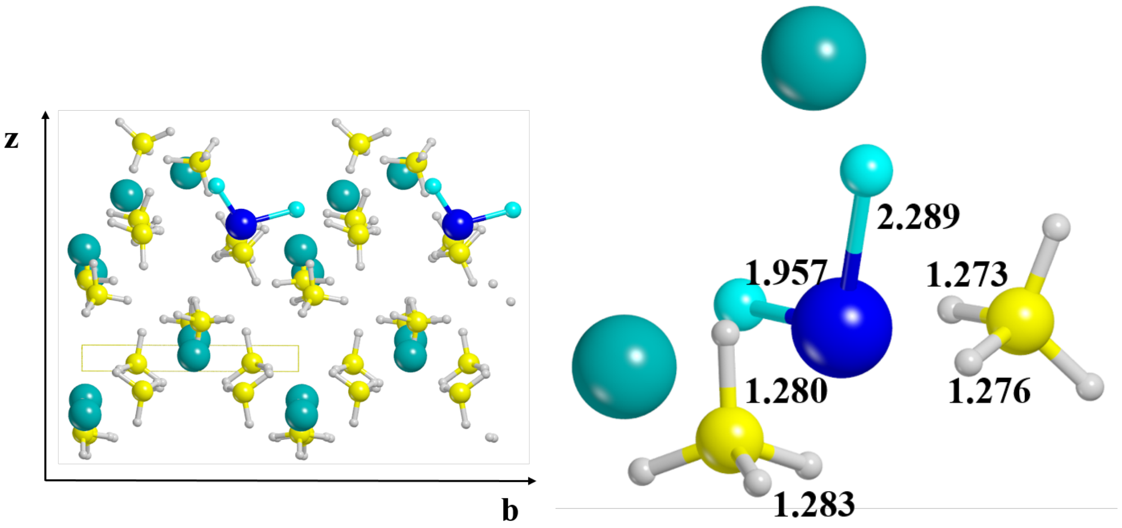

(a) Periodic structure

(b) Cluster

Figure S16: Periodic (left) and cluster (right) as extracted from the periodic structure.

Bond	Bond distance (Å)	
NiB	2.126	2.146
NiF	1.957	2.289
BH1	1.197	1.195
BH2	1.224	1.226
BH3	1.280	1.273
BH4	1.283	1.276

Table S9: Bond distances of the system doped with NiF_2 (high spin).

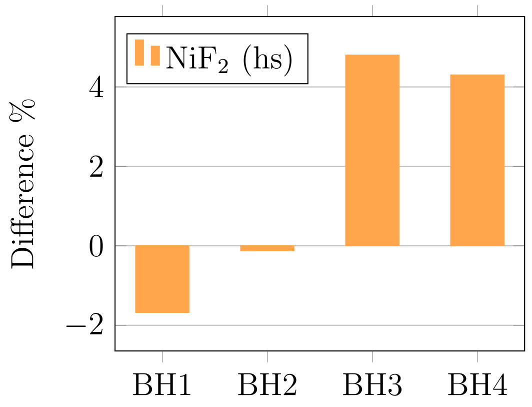

Figure S17: Percentage difference of B–H bond lenghts of the doped system respect to $Mg(BH_4)_2$ pure system.

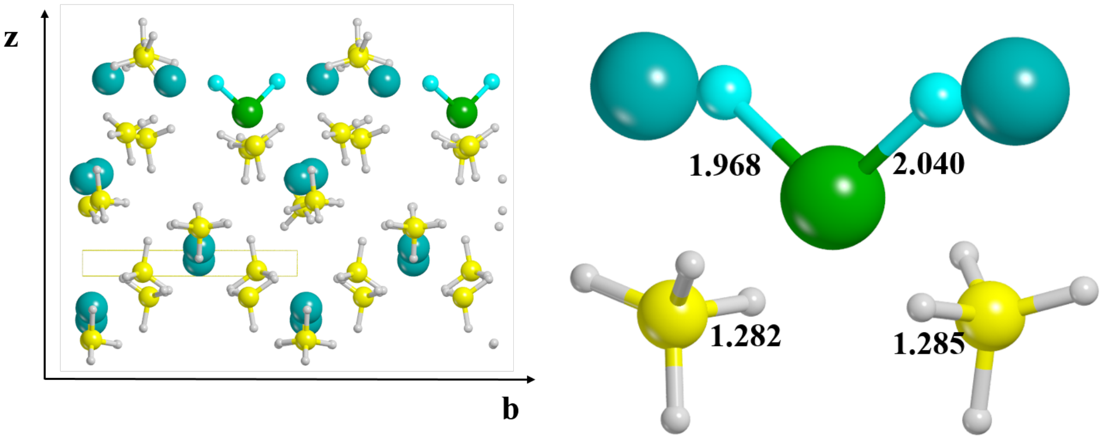

(a) Periodic structure (b) Cluster

Figure S18: Periodic (left) and cluster (right) as extracted from the periodic structure.

Bond	Bond distance (Å)	
NiB	2.328	2.308
NiF	1.968	2.040
BH1	1.204	1.214
BH2	1.232	1.219
BH3	1.237	1.223
BH4	1.282	1.285

Table S10: Bond distances of the system doped with CuF_2.

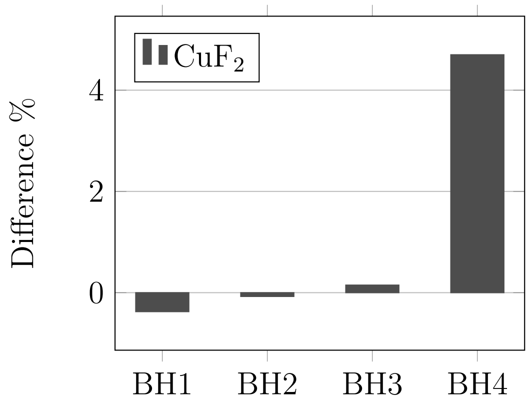

Figure S19: Percentage difference of B–H bond lenghts of the doped system respect to $Mg(BH_4)_2$ pure system.

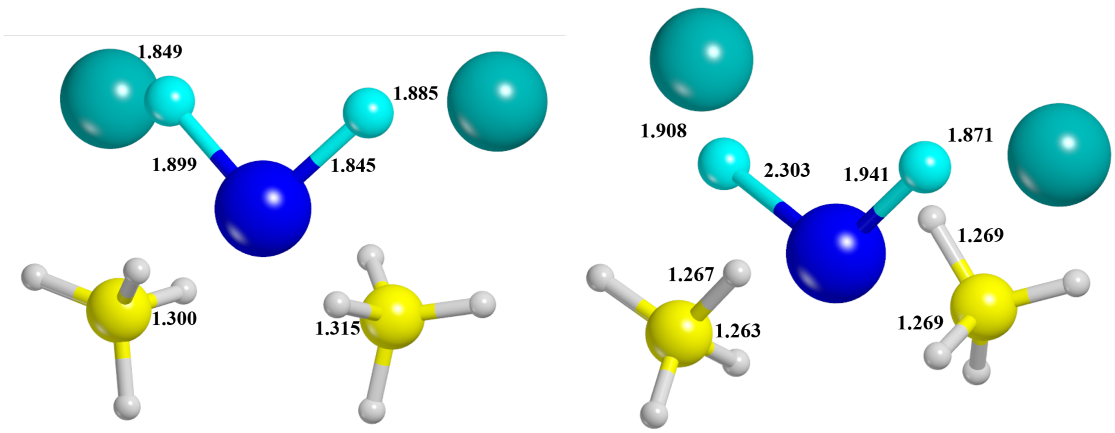

(a) M06-D* (ls) (b) M06-D* (hs)

Figure S20: Details of the NiF_2 doped $Pmc2_1$ $Mg(BH_4)_2$ (010) surface, optimized at the M06-D* level both in low (left) and high spin (right) configurations. Mg in cyan, Ni in blue, B in yellow, F in light blue, H in white.

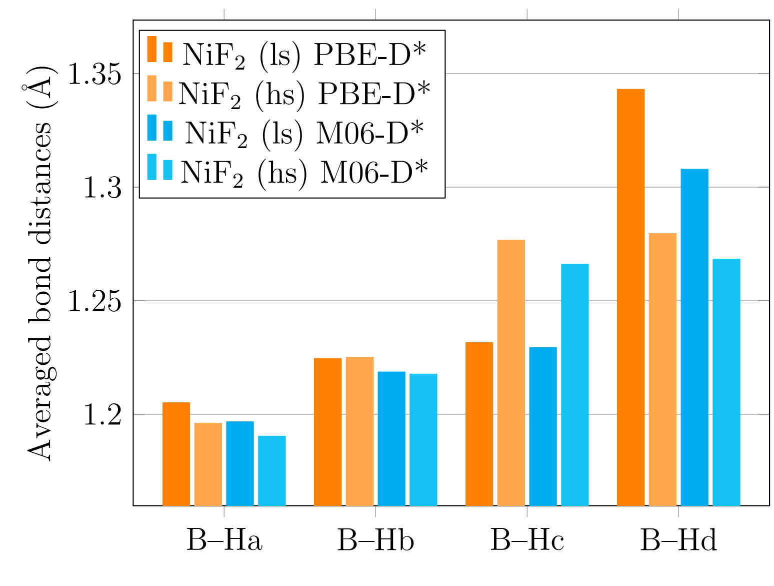

Figure S21: Average B–H bond distances (in Å) for the BH_4^- groups closest to the metal center, optimized at the M06-D* level.

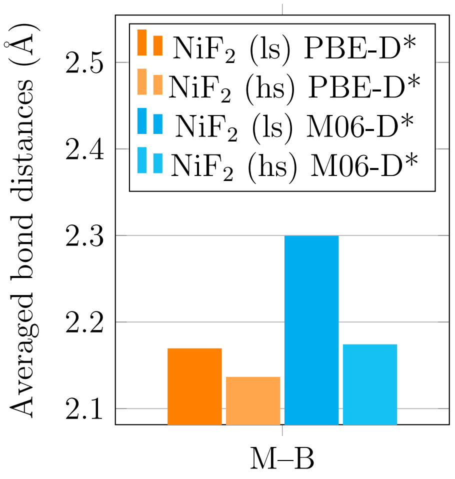

Figure S22: Average M–B bond distances (in Å) for the BH_4^- groups closest to the metal center, optimized at the M06-D* level.

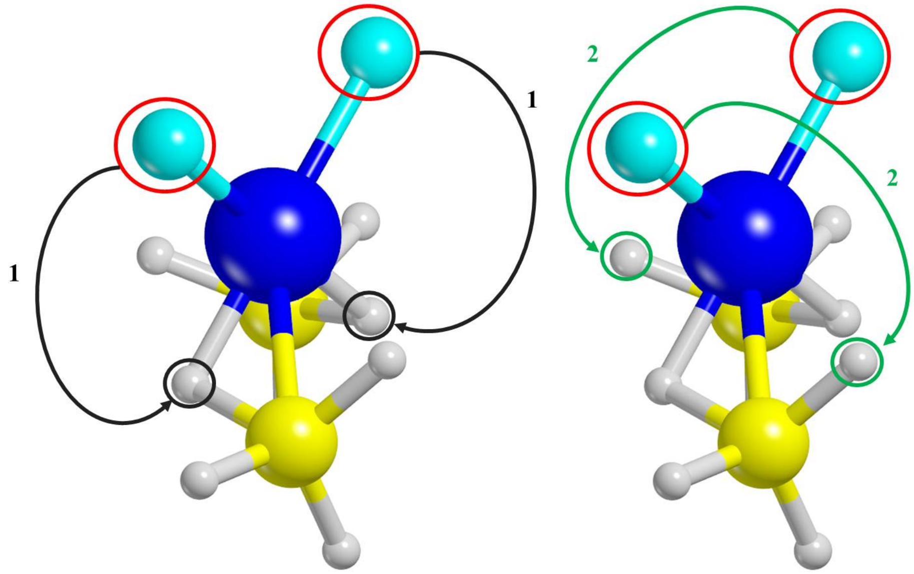

(a) Substitution 1 (b) Substitution 2

Figure S23: Clusters cut from the periodic structure with the substitutions from the reagent to Ni-P1 (left) and to Ni-P2 (right). Boron in yellow, nickel in blue, fluorine in cyan and hydrogen in white.

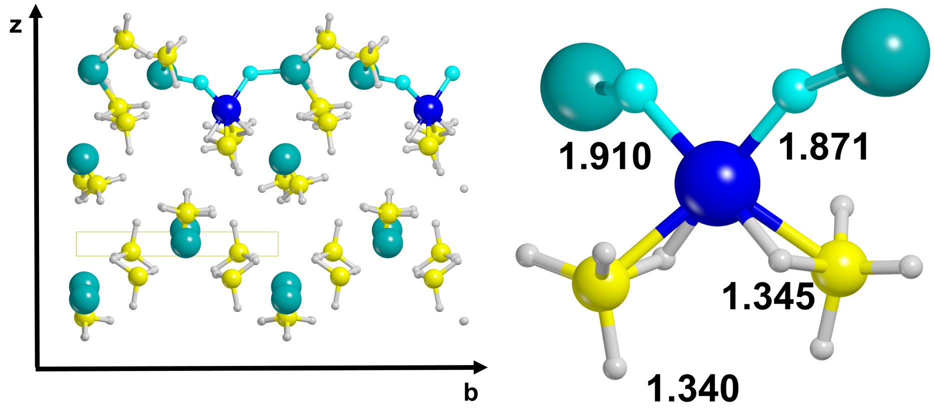

(a) Ni-R (0.0)

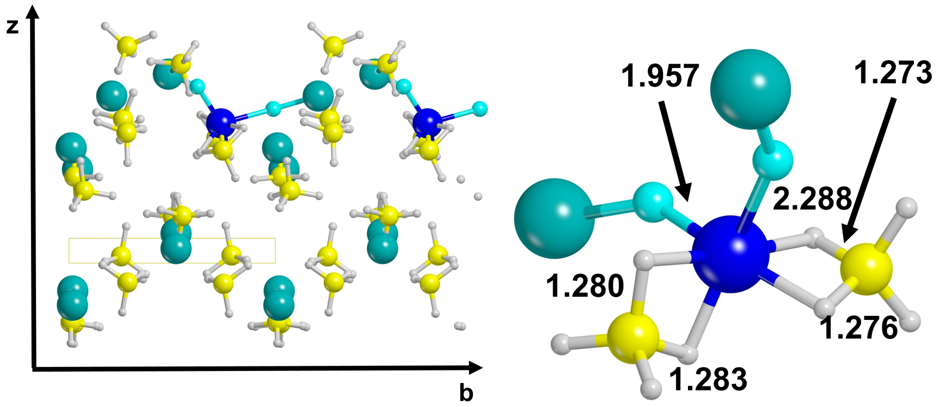

(b) Ni(hs)-R (-35.1)

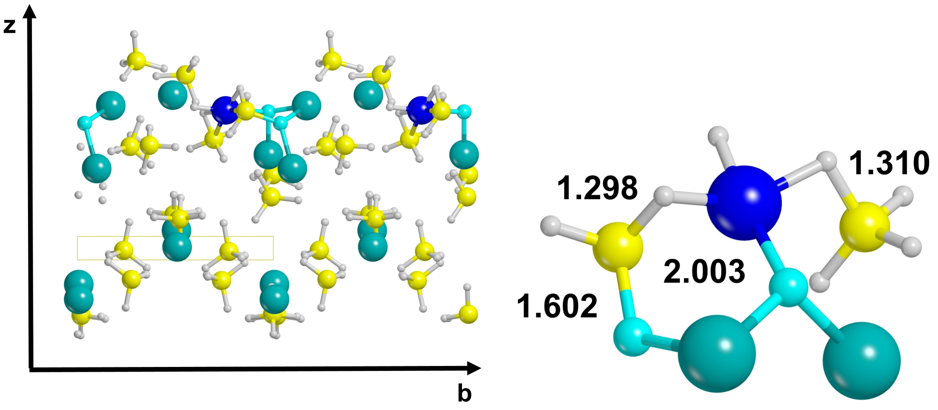

(c) Ni-I1 (-27.2)

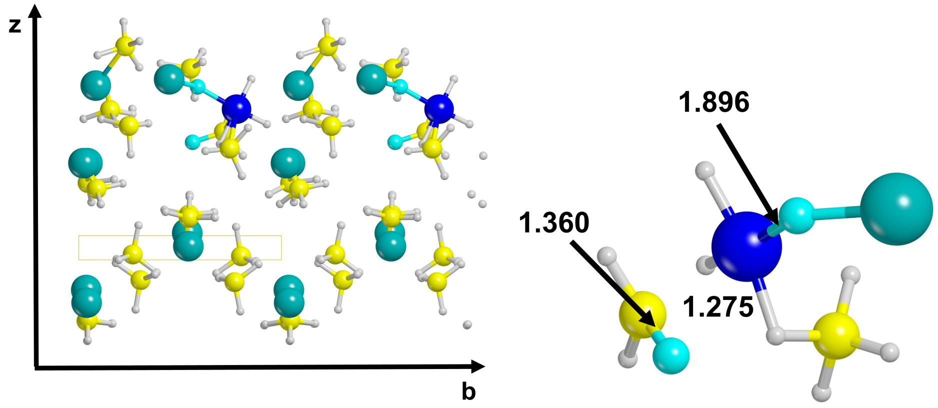

(d) Ni-I2 (48.0)

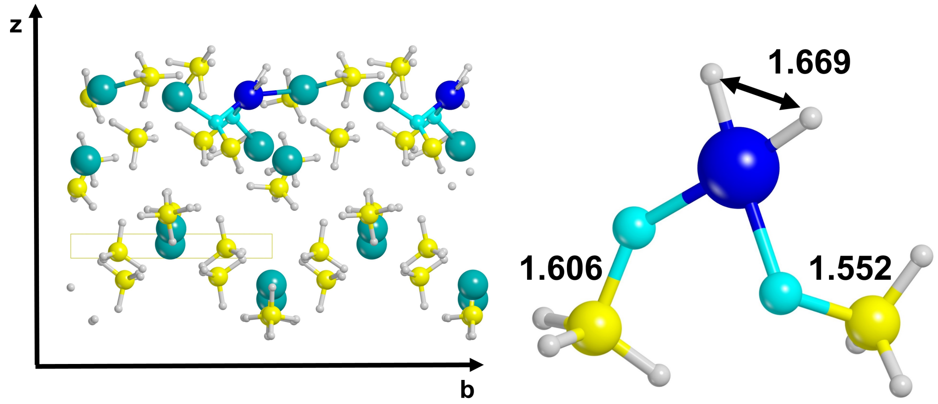

(e) Ni-P1 (104.6)

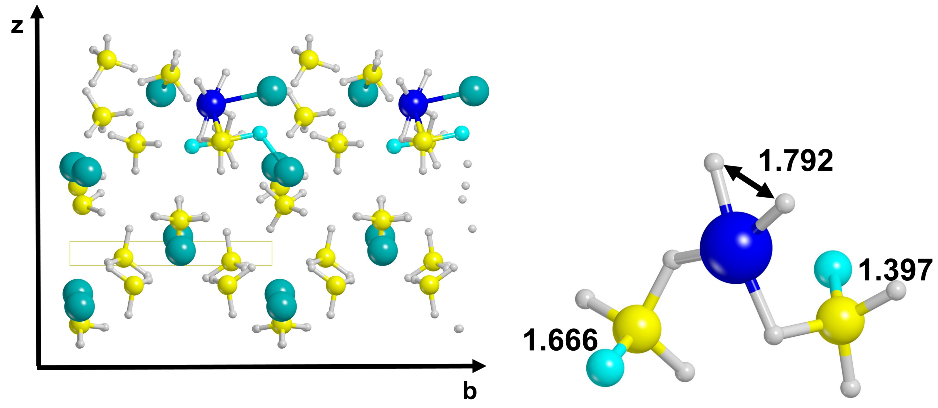

(f) Ni-P2 (76.9)

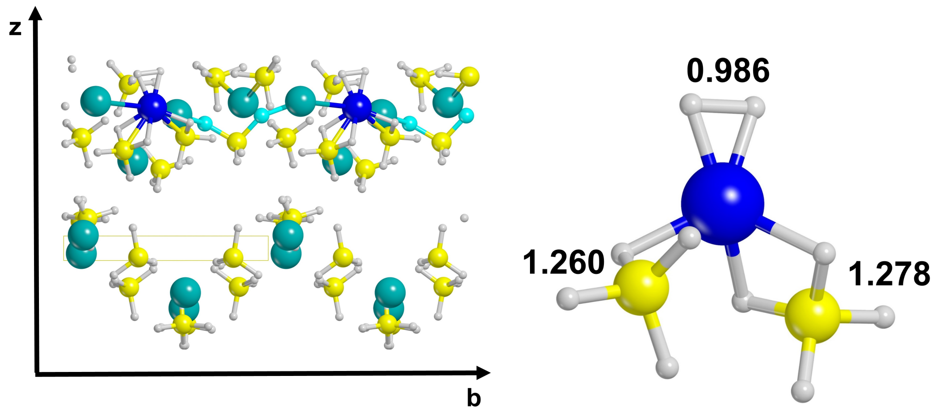

(g) Ni-P3 (-68.1)

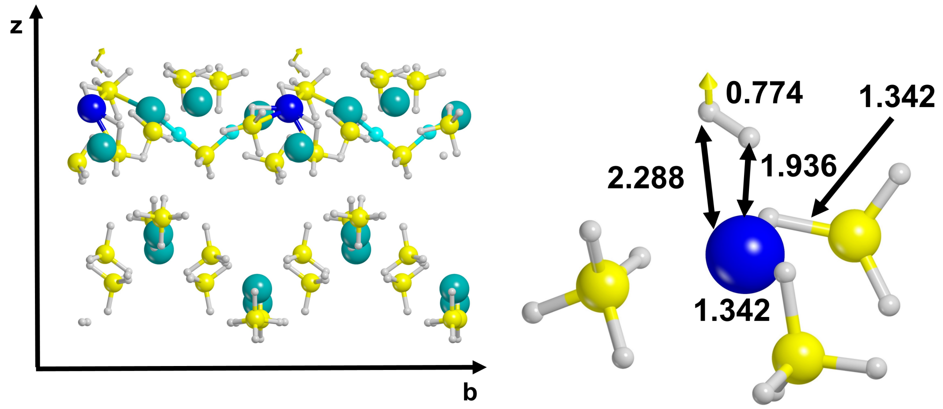

(h) Ni-TS (-31.6)

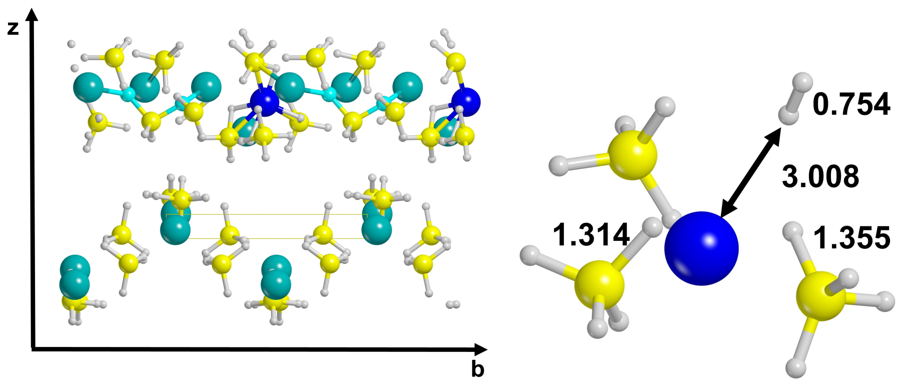

(i) Ni-P3-H_2 (-62.3)

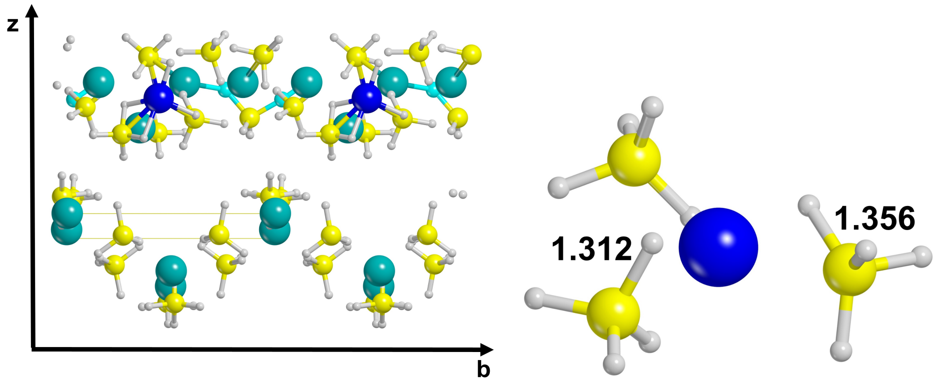

(j) Ni-P3 + H_2 (-58.4)

Figure S24: PBE-D* optimized structures for the doped NiF_2 (010) $Mg(BH_4)_2$ surface. Starting from the first figure, the following structures are reported: Ni-R, Ni(hs)-R, Ni-I1, Ni-I2, Ni-P1, Ni-P2, Ni-P3, Ni-TS, Ni-P3-H_2, and Ni-P3 + H_2. Bond distances are in Å. Energies are in kJ/mol. Boron in yellow, Magnesium in dark cyan, Nickel in blue, Fluorine in cyan and Hydrogen in white.

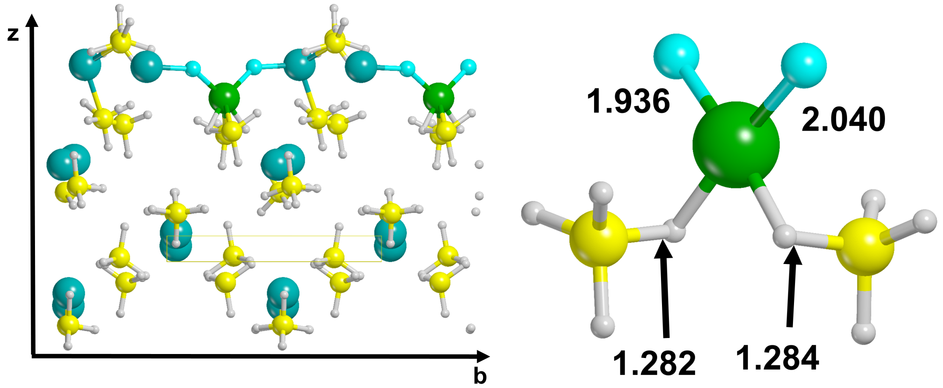

(a) Cu-R (0.0)

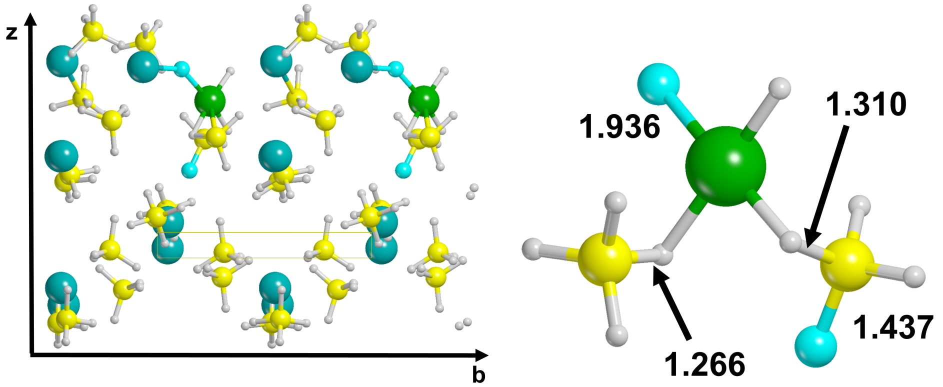

(b) Cu-I (60.8)

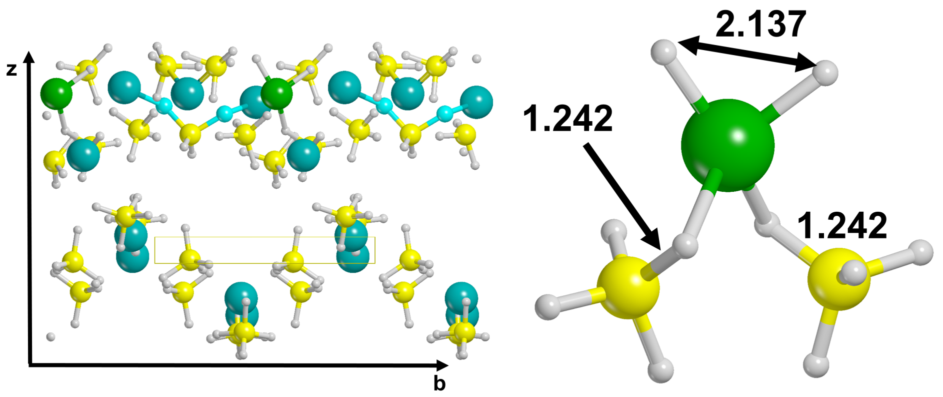

(c) Cu-P (18.6)

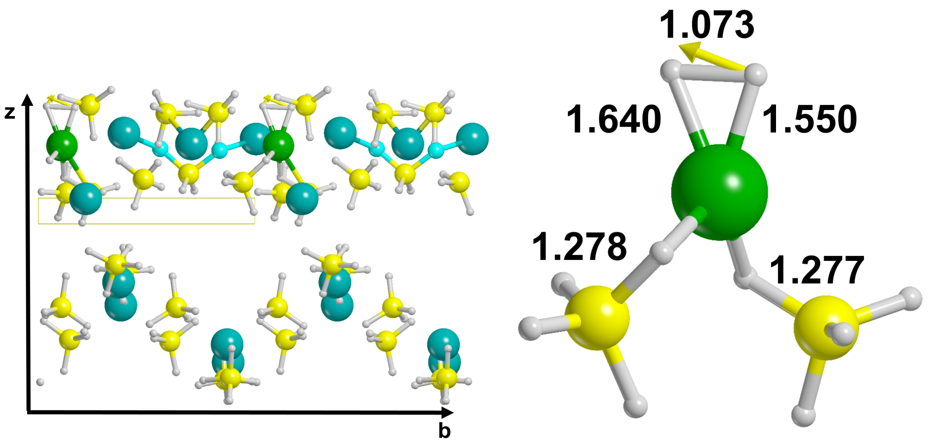

(d) Cu-TS (53.3)

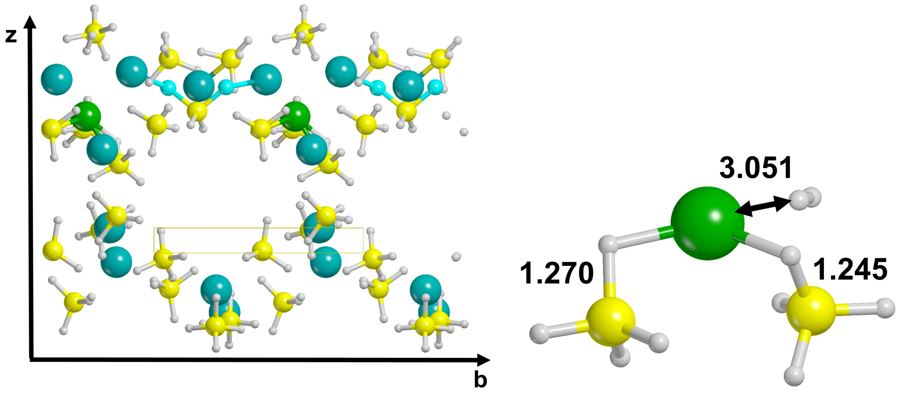

(e) Cu-P-H_2 (-4.2)

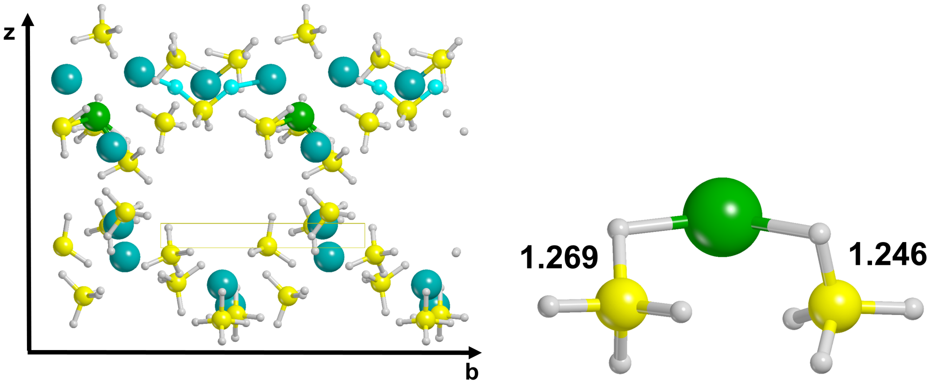

(f) Cu-P + H_2 (-1.0)

Figure S25: PBE-D* optimized structures for the doped CuF_2 (010) $Mg(BH_4)_2$ surface. Starting from the first figure, the following structures are reported: Cu-R, Cu-I, Cu-P, Cu-TS, Cu-P-H_2, and Cu-P + H_2. Bond distances are in Å. Energies are in kJ/mol. Boron in yellow, Magnesium in dark cyan, Copper in green, Fluorine in cyan and Hydrogen in white.
